# Investigation of the Robustness and Transferability of Adversarial Patches in Multi-View Infrared Target Detection

**DOI:** 10.3390/jimaging11110378

**Published:** 2025-10-27

**Authors:** Qing Zhou, Zhongchen Zhou, Zhaoxiang Zhang, Wei Luo, Feng Xiao, Sijia Xia, Chunjia Zhu, Long Wang

**Affiliations:** 1 School of Defence Science and Technology, Xi’an Technological University, Xi’an 710021, China; zhouqing@xatu.edu.cn (Q.Z.); xffriends@xatu.edu.cn (F.X.); 2 Unmanned System Research Institute, Northwestern Polytechnical University, Xi’an 710072, China; zhouzhongchen@nwpu.edu.cn; 3 Science and Technology Innovation Center, China Ship Development and Design Center, Wuhan 834099, China; 4 Aviation Engineering School, Air Force Engineering University, Xi’an 710038, China

**Keywords:** infrared images, adversarial examples, adversarial patches, target recognition

## Abstract

This paper proposes a novel adversarial patch-generation method for infrared images, focusing on enhancing the robustness and transferability of infrared adversarial patches. To improve the flexibility and diversity of the generation process, a Bernoulli random dropout strategy is adopted. The loss function integrates multiple components, including target hiding loss, smoothing loss, structural similarity loss, and patch pixel value loss, ensuring that the generated patches maintain low texture complexity and natural visual features. During model training, the Grad-CAM algorithm is employed to identify the critical regions of interest in the target detector, where adversarial patches are applied to maximize the attack effectiveness. Furthermore, affine transformations and random erasing operations are introduced to increase the diversity and adaptability of patches, thereby enhancing their effectiveness across different scenarios. Experimental results demonstrate that the proposed GADP (Generative Adversarial Patch based on Bernoulli Random Dropout and Loss Function Optimization) algorithm achieves a high Attack Success Rate of 75.8% on various target detection models, significantly reducing the average precision (AP). Specifically, the AP of the YOLOv5s model drops from 81.3% to 15.1%. Compared with existing adversarial attack methods such as advYOLO Patch and QR Attack, GADP exhibits superior transferability and attack performance, reducing the Average Precision of multiple detection models to around 40%. The proposed method is not only theoretically innovative but also shows potential practical value, particularly in tasks such as unmanned aerial vehicle (UAV) detection and ground security under low-visibility environments. This study provides new insights into adversarial attack research for infrared target recognition.

## 1. Introduction

Target detection under low-visibility conditions is a critical requirement for both UAV monitoring and ground-based security. In particular, human detection plays a vital role in scenarios such as nighttime urban security management, traffic control, and emergency scenario monitoring. Unlike visible-light imaging, infrared (IR) cameras can perceive human presence based on thermal radiation, thereby achieving stable and reliable detection performance in complex environments such as darkness, smoke, or haze. This advantage highlights the broad application potential of infrared human detection in monitoring systems deployed for ground security and UAV platforms. With the rapid advancement of deep learning techniques, deep-learning-based IR-based human detection has emerged as a key research focus in machine vision systems [1,2].

Although extensive research on adversarial examples has been conducted in the domain of visible-light images, studies focusing on infrared imagery remain relatively limited [3,4,5]. Similar to visible-light models, deep-learning-based infrared target detection models are also highly vulnerable to adversarial attacks. Due to the weaker texture and detail characteristics of infrared images, target features are more susceptible to perturbations [6], leading to reduced detection accuracy, false positives, or missed detections, which pose severe challenges for infrared-imaging-based remote sensing systems.

Adversarial examples typically appear in the form of pixel perturbations or adversarial patches [7,8,9]. Compared with pixel-level perturbations, adversarial patches are more feasible for real-world implementation, thus offering a practical advantage. In this attack paradigm, the adversary generates a small image region, referred to as an adversarial patch, and places it onto the target object so that the deep neural network misclassifies the target, yielding incorrect outputs [10,11]. More importantly, adversarial examples exhibit transferability, meaning that adversarial samples generated for one detection model can often successfully attack other models as well [12,13,14]. This phenomenon arises because, during feature extraction in infrared imagery, different models tend to focus on similar local characteristics around the target. Adversarial patches are able to disrupt these common features, thereby inducing bias across multiple detectors during feature extraction.

Adversarial sample attack methods can generally be categorized into white-box and black-box attacks. White-box attacks assume that the adversary has complete knowledge of the target model, including its architecture, weights, and internal parameters, which allows precise generation of adversarial perturbations [15,16,17]. In contrast, black-box attacks are carried out under the assumption that the adversary has no access to the internal details of the model and can only infer information based on input–output interactions [18,19,20]. In this study, we adopt the white-box attack setting, primarily because it leverages gradient information from the target model to efficiently generate adversarial examples and provides an upper bound for evaluating the effectiveness of attacks. Furthermore, white-box attacks enable the analysis of model robustness under extreme adversarial conditions, thereby offering valuable insights for the design of more resilient detection models.

In this paper, we propose a novel framework for generating adversarial patches in infrared (IR) target detection, termed GADP (Generative Adversarial Patch based on Bernoulli Random Dropout and Loss Function Optimization). The framework is designed with the primary goal of enhancing robustness and transferability while explicitly addressing the unique characteristics of infrared features and multi-view imaging. Unlike conventional adversarial patch-generation methods developed for visible-light scenarios, our approach fully accounts for the specific properties of IR imagery, including its single-channel nature, sparse texture information, and thermal radiation signatures of targets. Furthermore, a series of optimization strategies are incorporated into the algorithm to exploit these imaging characteristics effectively.

(1) We introduce a Bernoulli Stochastic Dropout (BSD) mechanism [21,22] during the patch generation process. By randomly discarding partial residual modules in the key feature-extraction stages of the backbone network, BSD simulates diverse feature distributions across different models. This strategy mitigates overfitting to a single detection model and significantly enhances the transferability of adversarial patches across different models. Compared with multi-model ensemble training, BSD does not substantially increase computational overhead while maintaining feature diversity and preserving detection accuracy.
(2) To address the challenges of physical deployment in infrared scenarios, we incorporate region constraints and robustness enhancement strategies. Specifically, we leverage Grad-CAM [23] to extract high-attention regions of the model and apply adversarial patches precisely to the decision-critical areas of the detector, thereby maximizing attack effectiveness within a limited perturbation range. Furthermore, we integrate data augmentation techniques such as affine transformations, scaling, and random erasing to simulate real infrared imaging conditions under varying viewing angles, distance changes, and partial occlusions. These strategies improve the adaptability of adversarial patches to complex scenarios such as drone-based aerial imaging and ground surveillance.
(3) In terms of loss function design, we construct a joint optimization objective comprising multiple constraints, including object-hiding loss, texture-smoothing loss, local structural similarity loss, and pixel-value constraint loss. This design not only ensures attack effectiveness but also enhances the naturalness and feasibility of the generated patches in infrared images. By reducing isolated noise points and maintaining plausible thermal radiation characteristics, the patches become more practical for real-world physical deployment.

Finally, to emulate application environments such as vehicle-mounted and UAV-mounted platforms, we conduct experiments on FLIR, LLVIP, and self-collected datasets to validate the effectiveness of the proposed method. We perform comprehensive comparisons of Attack Success Rates and transferability across various attack methods. The results demonstrate that adversarial patches generated by the GADP algorithm achieve superior performance on infrared imagery and consistently outperform current state-of-the-art methods. The main contributions of this study are as follows:

(1) This paper proposes a novel adversarial patch generation algorithm, GADP, which combines the Bernoulli Stochastic Dropout mechanism, loss function design, and robustness optimization strategies. The proposed method effectively attacks infrared image-based deep learning target detection models and demonstrates high robustness.
(2) By incorporating the random dropout technique, the transferability of adversarial patches across different target detection models is significantly improved. This reduces reliance on specific model architectures and enhances the general applicability and reliability of the attack.
(3) A loss function is designed that comprehensively considers object-hiding, smoothness, and structural similarity. This addition introduces quality constraints for the generated adversarial patches, ensuring their visual naturalness and effectiveness while minimizing the confidence of the target detector.
(4) By combining the Grad-CAM algorithm to identify critical regions and introducing affine transformations and random erasing strategies, the overfitting of patches to specific detection models is reduced. These strategies enhance the precision of adversarial attacks and improve the adaptability of the patches in varying environmental conditions.

## 2. Related Work

As stated in the introduction, this study focuses on white-box attack methods for infrared images. White-box attack techniques can generally be divided into three categories: (1) attacks based on generative adversarial networks (GANs), (2) attacks based on decision boundary analysis, and (3) attacks based on gradient optimization. In addition, patch-based attack methods constitute another important category, offering advantages for practical implementation in the real world. However, most of these methods have been specifically developed for image classification tasks in visible-light environments.

Among GAN-based attack approaches, generating adversarial examples using generative adversarial networks has proven to be an effective strategy. The joint optimization of the generator and discriminator is critical to this process; however, it also introduces instability during training, particularly when handling complex samples. Once trained, the generator can efficiently produce adversarial examples in batches, and these samples often demonstrate strong transferability. For instance, the AdvGAN method [24] achieved an Attack Success Rate of 92.76% on the MNIST challenge, with adversarial examples that are visually indistinguishable from real images. While effective in image classification tasks, this method shows only limited performance on high-resolution image classification and is generally unsuitable for complex object detection tasks.

Decision-boundary-based adversarial attacks primarily exploit the vulnerability of neural networks near decision boundaries by adding perturbations to push original samples closer to the boundary. Such methods are computationally efficient and capable of producing minimal perturbations. For example, Moosavi-Dezfooli et al. [25] proposed DeepFool, which precisely estimates the shortest distance from an input sample to the decision boundary and rapidly computes the corresponding minimal adversarial perturbation. Its efficiency and accuracy led to its widespread adoption in early research. Subsequently, the same authors introduced a universal perturbation algorithm [26], which extends this concept by aggregating multiple perturbation vectors, thereby systematically shifting a set of data points closer to the classifier’s decision boundary. Nevertheless, the decision boundaries of complex models are difficult to formulate explicitly. Consequently, such approaches are mainly applicable to relatively simple binary classification problems.

Gradient-based adversarial attack methods generate perturbations by computing the gradient of the loss function and applying them in the direction that leads the model to misclassify. The Fast Gradient Sign Method (FGSM) proposed by Goodfellow et al. [9] is a classical approach in this category. FGSM is highly efficient and can generate adversarial examples within a short time; however, the quality of the generated samples is often limited, making it unsuitable for more complex tasks such as object detection. In contrast, the Projected Gradient Descent (PGD) method proposed by Madry et al. [27] improves upon FGSM by introducing an iterative procedure. At each step, perturbations are carefully refined, resulting in significant improvements in attack accuracy. Despite being recognized as one of the most powerful attack methods, PGD requires substantial computational resources, and its adversarial examples tend to overfit specific model parameters, thereby reducing transferability. Carlini and Wagner (CW) [15] proposed an attack that minimizes the distance between adversarial examples and the original images while achieving a high misclassification rate. By adjusting confidence parameters, CW can bypass multiple defense mechanisms, and its small perturbations are advantageous for optimization. However, this method requires a binary search to tune hyperparameters of the loss function, making it relatively slow, especially when dealing with high-resolution images. Bubeck et al. [28] demonstrated that for any two-layer neural network, a single-step gradient descent is sufficient to identify adversarial examples. They further argued that universal adversarial perturbations exist with strong generalization ability, capable of degrading the performance of different models. Moreover, perturbations can also be generated along directions independent of specific input images. Dong et al. [29] introduced the Momentum Iterative Fast Gradient Sign Method (MI-FGSM), which accumulates gradients to achieve stable optimization and avoid local optima. By leveraging momentum techniques [30], MI-FGSM produces adversarial examples with superior performance compared to FGSM, although it has primarily been applied to image classification tasks.

In the domain of patch-based adversarial attacks, Liu X. et al. [31] proposed the DPatch method, which targets Faster R-CNN and YOLOv2 models by generating 40×40 small color patches to attack both bounding box localization and object classification. The adversarial patch paradigm facilitates practical deployment in the real world. Although effective against YOLOv2, DPatch fails to attack YOLOv3 and YOLOv4. Xie et al. [32] introduced the Dense Adversary Generation (DAG) method, which perturbs recognition models by optimizing a loss function. Specifically, it randomly replaces the original class and optimizes the score vector loss, ultimately driving model predictions away from the correct category. This approach has been applied to image segmentation and target recognition tasks. Adversarial perturbations in the physical world have also attracted significant attention [33,34]. Zhang et al. [4] proposed an adversarial patch optimization strategy for UAV remote sensing images, where a joint optimization scheme with scale factors enables the patch to maintain its attack effectiveness across multi-scale targets. Thys et al. [35] presented a printable adversarial patch, advYOLO Patch, that prevents YOLO9000 from detecting specific individuals. By generating perturbations against YOLOv2 and applying image enhancement to the patches, their method improved attack performance. However, the robustness of such patches is relatively weak, limiting their use to specific physical attack scenarios. Zhu et al. [36] proposed the QR Attack, which demonstrates that adversarial “QR code” patterns can be designed to evade infrared detectors effectively from multiple viewing angles when applied to infrared-adversarial clothing. Sharif et al. [34] designed a pair of adversarial eyeglasses to fool face recognition systems, while Evtimov et al. [37] misled traffic sign recognition models by attaching black-and-white stickers to road signs. More recently, Hu et al. [10] proposed the Target-Centric Expandable Generative Attack (TC-EGA), which generates adversarial patches capable of misleading models under varying camera viewpoints. This method trains a generator to produce patches based on random vectors but suffers from poor transferability, being effective only against YOLOv2, YOLOv3, and Faster R-CNN in attacking human targets.

In summary, a comparison of adversarial attack methods reported in the literature is presented in Table 1.

Among the above adversarial attack approaches, patch-based strategies exhibit strong potential for real-world applications. To further advance this direction, this paper proposes a novel adversarial patch-generation method tailored for infrared target detection. Our approach focuses on enhancing the robustness and transferability of adversarial patches in infrared imaging while accounting for the unique characteristics of infrared sensors. By integrating a series of optimization strategies, the proposed method generates adversarial patches capable of maintaining effective attack performance in complex surveillance scenarios.

## 3. Method

### 3.1. Adversarial Attack Framework

**Figure 1:**
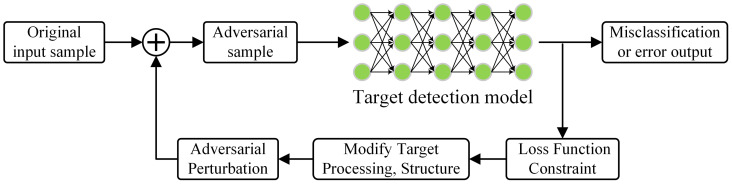
Principle of traditional adversarial attack methods.

For object detectors, the principle of traditional adversarial attack methods is illustrated in Figure 1.

Deep-learning-based target recognition networks optimize the loss function through iterative training to improve object detection accuracy. When adversarial perturbations are applied to the object detector, the adversarial attack reduces the confidence of the detector in the target, potentially leading to the target being filtered out, resulting in missed detection.

This paper proposes a novel adversarial attack method for infrared images using a new intelligent recognition algorithm. Let the input infrared image be denoted as *x*, with the original infrared image distribution represented as *D*, which contains one or more instances of “human” or similar targets, i.e., x∈D. The pre-trained infrared object detector f:x→Z predicts the target label $Z^{\prime }$ for the input infrared image. The label $Z^{\prime }$ is expected to match the real label *Z* of the infrared image with high accuracy, where *Z* includes the bounding box position $l_{\mathrm{pos}}$, the probability $p_{\mathrm{obj}}$ that the object is in the foreground, and the class score $p_{\mathrm{cls}}$.

(1) $$Z^{\prime }:=\left[l_{pos},p_{obj},p_{cls}\right]=f\left(x\right)$$

The objective of the proposed adversarial attack algorithm is to prevent the infrared object detector from recognizing humans, i.e., $p_{\mathrm{obj}}=0$. We adopt a patch-based adversarial attack strategy in which adversarial patches replace local regions of the original infrared images. The overall objective can be formulated as follows:

(2) $$\arg\min p_{obj}=\arg\min_{i}f\left(x^{adv}\right)$$

where $x^{adv}$ denotes the adversarial example obtained by adding an adversarial patch δ to the original input infrared image *x*. The index of the *i*-th image in the distribution *D* is denoted as *i*.

**Figure 2:**
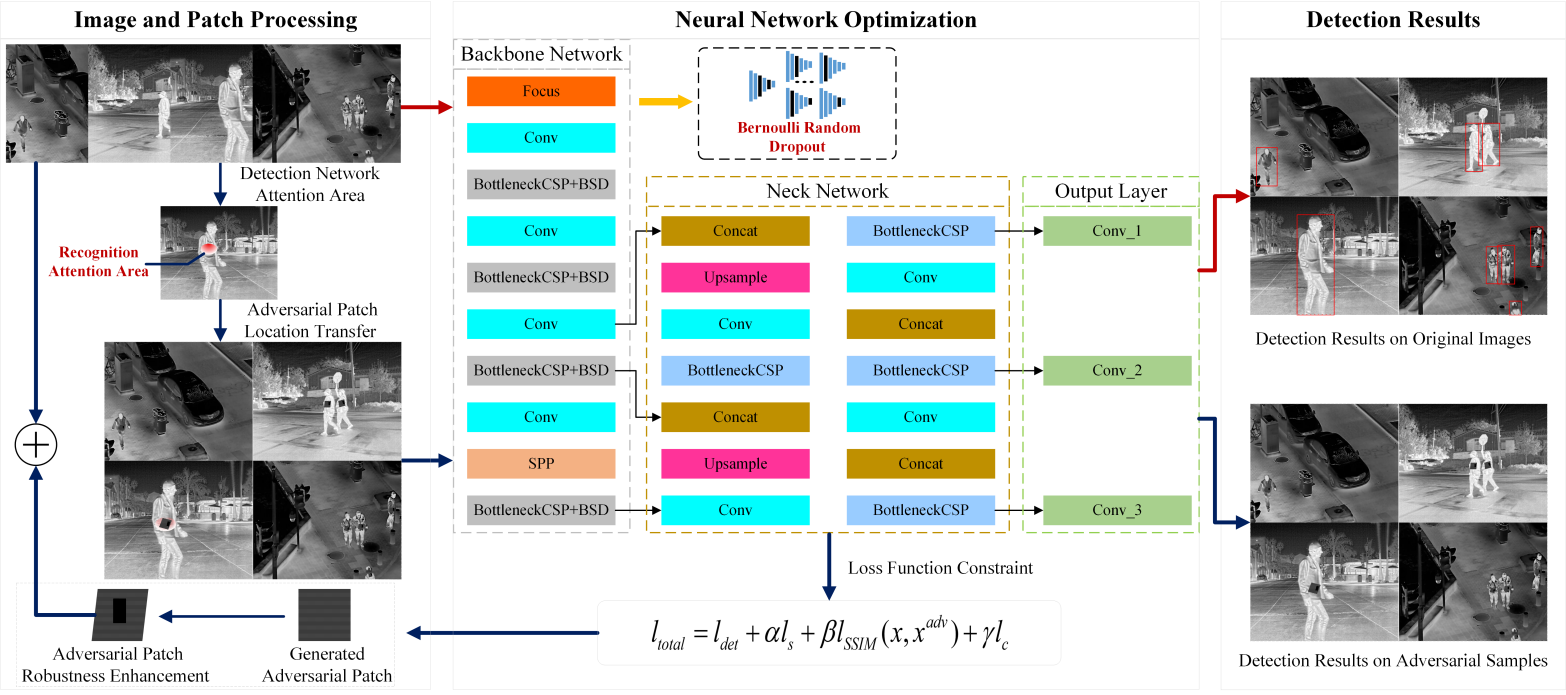
Framework of the proposed GADP algorithm.

This study focuses on enhancing the robustness and transferability of adversarial patches in infrared target detection. To this end, we propose a Generative Adversarial Patch algorithm based on Bernoulli Random Dropout and Loss Function Optimization (GADP). The overall framework of the proposed GADP algorithm is illustrated in Figure 2.

During the data preprocessing stage, patch augmentation was performed to improve the robustness of the adversarial patches. Furthermore, by identifying the attention regions of the target recognition neural network that correspond to critical target features, the adversarial patches were precisely positioned to interfere with these important features. In the design of the deep neural network, the Backbone is responsible for feature extraction. The introduction of Bernoulli Stochastic Dropout (BSD) enhances model robustness, mitigates overfitting, and improves the transferability of adversarial patches. The Neck is employed for multi-scale feature fusion; at this stage, introducing BSD randomly discards a portion of the features, which interferes with feature transmission. The Head performs the final classification and regression tasks, which require complete feature information. Therefore, randomly discarding features at this stage would result in information loss. Consequently, BSD is applied exclusively to the CSP modules in the backbone. This strategy enhances the generalization and transferability of the model without compromising feature fusion or detection performance. In addition, by constraining the adversarial loss function, the generated adversarial patches are better adapted to infrared imaging scenarios, thereby improving the success rate of the attack algorithm. As illustrated in Figure 2, the upper-right image shows the detection results on the original sample, while the lower-right image shows the detection results on the adversarial sample, where the attack causes the model to fail to detect the target. The pseudocode of the proposed GADP algorithm is provided in Algorithm 1 as follows:

The aforementioned algorithm generates adversarial patches through iterative optimization, enabling them to effectively attack infrared object detectors under various viewpoints, scales, and partial occlusions. To rigorously define this optimization process, a complete mathematical formulation is presented in the following section.
**Algorithm 1:** GADP Adversarial Attack Algorithm
Input: Original input sample images: $x_{1}$, $x_{2}$, *…*, $x_{N}$, The target infrared object detection            model *D*, number of iterations *E*, batch size *B*, initial patch ${\mathrm{patch}}_{0}$
Output: Optimized adversarial patch


Initialize the value of patch as ${\mathrm{patch}}_{0}$
For e∈[0,E) do:
       Input the batch of original infrared images into model *D*, obtaining the target
        bounding box positions and confidence scores;
       Identify the neural network attention regions corresponding to the target in the         infrared image;
       Apply the transformed adversarial patch to the original infrared image, incorporating         the attention regions, generating the adversarial sample $x_{\mathrm{adv}}$;
       Apply Bernoulli random dropout to the backbone network of model *D*;
        Obtain the target bounding box confidence and location information for the adversarial       sample $x_{\mathrm{adv}}$ in model *D*;
       Compute the loss function value;
       Update the adversarial patch using the Adam optimizer based on the gradient of
       the loss function: $\mathrm{patch}\leftarrow \mathrm{Adam}(\mathrm{patch},\nabla_{\mathrm{patch}}L)$
       Perform affine transformation and random erasing on the adversarial patch;
       Obtain the transformed adversarial patch;
End for
Obtain the transformed adversarial patch.


### 3.2. Optimization Problem Formulation

Based on the attack objective in Equation (2), we formulate the adversarial patch generation problem as the following constrained optimization problem:

(3) $$\begin{array}{cc} P^{*} & =\arg\min_{P}E_{x_{i}∼D,T,M}\left[P_{obj}\left(f_{M}\left(x_{i}^{adv}\right)\right)\right]=\arg\min_{P}E_{x_{i}∼D,T,M}\left[L_{total}\left(f_{M}\left(x_{i}^{adv}\right),Z_{i}\right)\right]\\ \; & \mathrm{subject}\;\mathrm{to}\;p_{obj}\left(f_{M}\left(x_{i}^{adv}\right)\right)\to 0,P\in C \end{array}$$

where $x_{i}^{adv}=x_{i}⊙A\left(P,T,\left(x_{p},y_{p}\right)\right)$ denotes the adversarial sample obtained by applying the adversarial patch to the original image $x_{i}$.

The objective of this optimization problem is to find the optimal adversarial patch $P^{*}$ such that when applied to images from the data distribution D, the detector outputs the target confidence $p_{obj}$ that is minimized, thereby achieving objective leakage. The optimization process directly implements the suppression of $p_{obj}$ by minimizing the expected loss function $E[L_{total}]$. The expected operation here is performed simultaneously across three dimensions: the image distribution D (ensuring effectiveness across different images), the random transformation parameters T (enhancing robustness to viewpoint and scale variations), and the adversarial random dropout strategy M (improving adaptability to partial occlusions during the adversarial perturbation extraction process). The detector $f_{M}$ represents the model after applying the BSD mechanism during inference, where the outputs of certain convolutional layers are stochastically dropped via the adversarial dropout distribution.

The adversarial sample $x_{i}^{adv}$ is generated by the function A. This function first applies a random affine transformation T to the patch *P* (including rotation and scaling) and then attaches it to the specified location $\left(x_{p},y_{p}\right)$ on the original image $x_{i}$. The location parameters $\left(x_{p},y_{p}\right)$ are determined by Grad-CAM attention region analysis. The annotation *Z* contains the ground-truth bounding boxes and class information for all human targets in the *i*-th image, which are used to calculate the loss function.

The constraint set C encompasses three types of constraints: (1) pixel value range constraints, ensuring the physical printability of the patch; (2) position and shape constraints, based on Grad-CAM to determine the attachment location and size; (3) transformation parameter constraints, enhancing robustness through affine transformations and random erasure. The specific forms and parameter settings of these constraints are detailed in Section 3.5 and Section 3.6. The loss function $L_{total}$ comprehensively considers attack effectiveness, visual naturalness, and physical realizability. The core term $L_{hide}$ directly acts on the target confidence $p_{obj}$, while auxiliary terms include smoothness loss, structural similarity loss, and pixel distribution loss, jointly ensuring that the generated patch does not introduce visual anomalies during physical applications. The specific definitions and weight settings for each loss term are provided in Section 3.4.

The optimization process employs Stochastic Gradient Descent (SGD), where in each training batch, random images $x_{i}∼D$ are sampled, and the transformation parameters T and BSD strategy M are applied. The patch *P* is then updated via backpropagation. This stochastic strategy enhances the robustness of the generated patch $P^{*}$. The overall optimization procedure is presented in Section 3.1.

To elucidate the theoretical foundation of the proposed method, we compare Equation (3) with the classical PGD attack [27]. The optimization objective of PGD is

(4) $$\delta^{*}=\arg\;\max_{{∥\delta ∥}_{\alpha }\leq \epsilon }L\left(f\left(x+\delta \right),y_{\mathrm{true}}\right)$$

where δ is the global perturbation, achieved through iterative projected gradient ascent:

(5) $$\delta_{t+1}=\Pi_{{∥\delta ∥}_{\alpha }\leq \epsilon }\left(\delta_{t}+\alpha \cdot \mathrm{sign}\left(\nabla_{\delta }L\right)\right)$$

This paper differs from PGD in the following three key respects. Attack form: PGD generates full-image perturbations δ, while this paper generates localized patches *P*. The patches can be printed and physically attached to objects in the real world, offering stronger physical realizability. Optimization objective: PGD aims to maximize classification loss, while this paper aims to directly minimize detection confidence $p_{obj}$, achieving stealthier and more effective adversarial attacks. Regularization mechanism: PGD constrains the perturbation magnitude through the $L_{\infty }$ norm, while this paper imposes cross-modality constraints via T and M to enhance physical robustness (BSD mechanism) simultaneously. From a mathematical perspective, PGD seeks the perturbation for a single image–model pair:

(6) $$-\min_{\delta }L\left(f\left(x+\delta \right),y\right)\;s.t.\;{∥\delta ∥}_{\infty }\leq \epsilon$$

This paper seeks the cross-distribution, cross-transformation, and cross-model regularization of patch *P*:

(7) $$\min_{P\in C}E_{x_{i}∼D,T,M}\left[L_{total}\left(f_{M}\left(x_{i}^{adv}\right),Z\right)\right]$$

embedding the regularization constraints into the optimization framework.

### 3.3. Adversarial Patch Transferability and Enhanced Strategies

#### 3.3.1. Theoretical Foundation of Transferability

The transferability of adversarial patches across different model types is a critical discovery in adversarial attack research, which has been extensively validated experimentally [12,13,14]. However, due to the black-box nature and inherent non-differentiability of deep neural networks, the intrinsic mechanisms underlying patch transferability remain without a unified mathematical framework. Among these, the most classical approach is based on the gradient similarity theory [9]. This theory posits that when different models are trained on similar data, their loss surfaces exhibit certain correlations, which can be expressed as

(8) $$\delta^{*}=\arg\;\max_{{∥\delta ∥}_{\infty }\leq \epsilon }E_{M∼M}\left[L_{M}\left(x+\delta ,y\right)\right]$$

where M denotes the model ensemble, $L_{M}$ represents the loss function of model *M*, *x* is the input sample, *y* is the ground truth label, and ϵ is the perturbation magnitude. This formula demonstrates that the maximum likelihood perturbation expected over multiple model loss functions exhibits robust transferability across adversarial attacks.

Based on this finding, the internal mechanisms of transferability are not the primary focus of this study. This paper instead concentrates on enhancing the transferability of adversarial patches across different detection models through the proposed Bootstrap Selective Dropout (BSD) strategy. By introducing model diversity during the training process, we further strengthen the patch’s transfer capability across heterogeneous detection models.

#### 3.3.2. Bernoulli Stochastic Dropout

Enhancing the transferability of adversarial patches improves the effectiveness of attacks across diverse target recognition models, thereby reducing the reliance of attackers on the parameters and architectures of a specific model. Moreover, high transferability increases the stealthiness of the attack, making it more resistant to detection by defense systems and ultimately improving the overall success rate. A straightforward approach to improving the transferability of adversarial patches is to jointly train the patches with an ensemble of multiple-target-recognition models. However, such an approach often results in excessively complex models, incurs substantial computational overhead, and may introduce interference among models, which makes it difficult to obtain optimal adversarial patches. To overcome these limitations, we design a Bernoulli Stochastic Dropout method, which incorporates a local masking strategy into adversarial patches. The details of this method are presented below.

Random depth [21] is an algorithm designed to reduce the effective depth of a network during training while keeping the network depth unchanged during testing. Specifically, this algorithm randomly removes entire residual blocks during training, and the deleted blocks are bypassed through skip connections. The formulation is given as follows:

(9) $$H_{l}=\mathrm{RELU}\left(T_{l}f_{l}\left(H_{l-1}\right)\right)+id\left(H_{l-1}\right)$$

The Bernoulli random variable $T_{l}\in \left\{0,1\right\}$, where id(·) denotes the identity mapping. When $T_{l}=1$, the *l*-th residual block is activated; when $T_{l}=0$, the *l*-th residual block is discarded. The output before the *l*-th residual block is $H_{l-1}$, and the output after the *l*-th residual block is $H_{l}$. By multiplying the function $f_{l}$ by the Bernoulli random variable, the algorithm effectively skips the *l*-th residual block in the neural network. However, discarding too many shallow layers can cause the model to lose important input features, resulting in a drop in accuracy. The random depth algorithm integrates a linear decay rule and defines the survival probability of the *l*-th residual block as follows:

(10) $$p_{l}=P\left(T_{l}=1\right)=1-\frac{l}{L}\left(1-p_{L}\right)$$

The survival probability of a residual block is computed based on the current layer *l*, where the survival probability decreases with increasing layer depth. The survival probability of the input layer is $p_{0}=1$, meaning it will not be discarded, while the survival probability of the last residual block is $p_{L}$.

**Figure 3:**
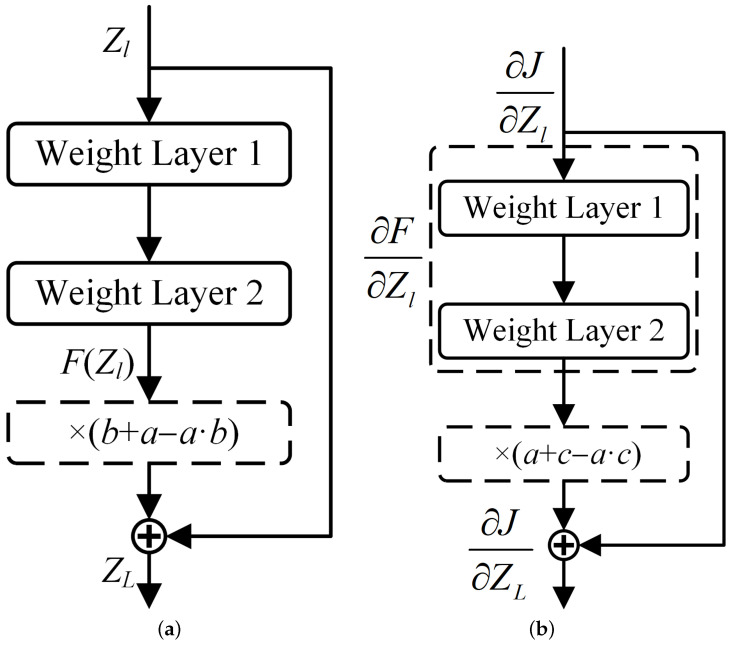
Schematic of the Bernoulli Stochastic Dropout. (**a**) Forward Propagation; (**b**) Backward Propagation.

Inspired by the random depth algorithm, this chapter introduces the Bernoulli Stochastic Dropout method. In the Bernoulli random dropout method, we have D∼Bernoulli(p), where *p* represents the dropout probability. By randomly discarding certain layers of the neural network, this method allows for the random retention or dropping of features during training, which increases feature diversity and enhances the robustness of the generated adversarial patch against adversarial attacks. As a result, the patch exhibits stronger adaptability to various target recognition models. Simultaneously, the random dropout strategy effectively reduces the risk of overfitting, making the adversarial patch more flexible when applied to new models. Furthermore, simulating the behavior of different models brings about the effect of ensemble learning, further improving the stability and performance of the adversarial patch under different conditions. The schematic diagram is shown in Figure 3:

In the forward propagation process, the Bernoulli Stochastic Dropout method inputs $Z_{l}$ of the CSP module and the stacked layer output $F(Z_{l})$ of the residual block to obtain the output $Z_{L}$. Specifically, *a* is sampled from a continuous uniform distribution U(0,1), and *b* is sampled from a Bernoulli distribution. The formula is as follows:

(11) $$Z_{L}=Z_{l}+\left(b+a-a\cdot b\right)\cdot F\left(Z_{l}\right)$$

Here, (b+a−a·b) is a composite random variable. When b=1 (no dropout), this term equals 1, and the residual path is fully preserved; when b=0 (dropout occurs), this term equals a∈(0,1), and the residual path is partially suppressed but not completely eliminated. This design ensures that even in the dropout state, gradients can still propagate in an attenuated form, thereby preventing the vanishing gradient problem.

During backpropagation, the loss function is defined as *J*. According to the chain rule, the gradient with respect to the CSP module input $Z_{l}$ is calculated as

(12) $$\psi \left(\frac{\partial J}{\partial Z_{l}}\right)=\psi \left(\frac{\partial J}{\partial Z_{L}}\cdot \frac{\partial Z_{L}}{\partial Z_{l}}\right)=\frac{\partial J}{\partial Z_{L}}\left(1+\left(c+a-a\cdot c\right)\cdot \frac{\partial F}{\partial Z_{l}}\right)$$

where *c* is a Bernoulli variable with the same distribution as *b* but sampled independently. This formula indicates that gradient backpropagation is similarly affected by random dropout; however, due to the presence of the uniform distribution variable *a*, the gradient never completely becomes zero, ensuring training stability. Mathematically, the expected value is E[(b+a−a·b)]=p+(1−p)·0.5=p+0.5(1−p), which guarantees the statistical balance of the dropout operation.

By applying this method to randomly drop partial residual paths in the CSP modules of the object detector’s backbone network, we effectively mitigate overfitting and vanishing gradient issues in deep neural networks. Since BSD only affects the CSP modules without impacting the neck and head networks, the model maintains its full feature transmission capability while achieving an implicit ensemble learning effect by introducing randomness during the feature-extraction stage. This approach trains multiple randomly dropped sub-models, and the reduction in model layers can accelerate the training speed.

For the same input $Z_{l}$, the randomness of BSD causes the output $Z_{L}$ to become a random variable. According to Equation (11), the expected characteristic variance is defined as

(13) $$\sigma_{\mathrm{feat}}^{2}={\mathrm{Var}}_{b,\sigma }\left(Z_{L}\right)=E_{b,\sigma }\left[{\left(Z_{L}-E\left[Z_{L}\right]\right)}^{2}\right]$$

In this work, we obtained an expected characteristic variance of 0.125 after applying BSD, compared to the baseline characteristic variance of 0.075 without BSD, showing a significant increase. This demonstrates that the BSD method effectively enhances the diversity of feature representations. We further employ Shannon entropy to evaluate the information diversity of the features, with the formula for feature entropy given as

(14) $$H\left(Z_{L}\right)=-\sum_{i=1}^{D}p_{i}\log p_{i}$$

where $p_{i}$ is the normalized probability of the *i*-th feature dimension, and *D* is the feature dimensionality. In our experiments, the feature entropy increased from H=1.83 to H=2.16 (+18.0%), validating that BSD significantly enhances the diversity of feature extraction.

### 3.4. Loss Function for Adversarial Attack Algorithm

#### 3.4.1. Total Loss Function

During the generation of adversarial patches, the loss function is used to measure the difference between the target detector’s prediction and the desired output. Unlike visible light images, infrared images are based on thermal radiation for imaging, making it more challenging to present complex textures in the physical world. Therefore, during patch generation, we aim to generate patches with relatively fewer patterns.

To ensure that the infrared target recognition model fails to recognize the target, the GADP adversarial attack algorithm proposed in this paper ensures a significant deviation between the desired output and the ground truth, such that the desired output does not contain the specified target class. To optimize both the texture and image information of the adversarial patch, constraints are applied to the loss function to ensure that the target recognition model cannot identify the target. The total loss function is given by

(15) $$L_{\mathrm{total}}=\lambda_{1}L_{\mathrm{hide}}+\lambda_{2}L_{\mathrm{smooth}}+\lambda_{3}L_{\mathrm{SSIM}}+\lambda_{4}L_{\mathrm{pixel}}$$

where $\lambda_{1}$, $\lambda_{2}$, $\lambda_{3}$, and $\lambda_{4}$ are weighting coefficients corresponding to the hiding loss $L_{\mathrm{hide}}$, smoothness loss $L_{\mathrm{smooth}}$, structural similarity loss $L_{\mathrm{SSIM}}$, and pixel value constraint loss $L_{\mathrm{pixel}}$, respectively. To ensure attack effectiveness while maintaining visual naturalness of the image, the selection of these weighting coefficients in the loss function must balance the trade-off between Attack Success Rate (ASR) and image quality metrics (such as SSIM and subjective visual evaluation).

To ensure the loss function effectively balances multiple optimization objectives, we adhere to the magnitude balance principle, maintaining the weighted loss terms within comparable orders of magnitude to guarantee gradient stability and training convergence. Building upon this foundation, and recognizing that target hiding constitutes the core attack task of this work, demanding higher priority, we allocate the target hiding term to contribute 60–70% of the total loss (dominant role). The smoothness constraint is critical for physical-world robustness and is thus assigned 10–20% (physical robustness). Pixel feasibility serves as a necessary condition for physical attacks, accounting for 10–20% (physical realizability). Given that infrared images inherently exhibit lower contrast, overly strong SSIM constraints would limit the optimization space; therefore, we allocate 5–10% to this term (visual naturalness). The specific weight values are derived through magnitude analysis of each loss component (detailed in Section 3.4.3) and formal derivation (presented in Section 3.4.4).

#### 3.4.2. Detailed Definition of Each Loss Term

To prevent the target detector from recognizing the target, the adversarial attack algorithm aims to reduce the target object’s confidence to zero, guiding the network to predict the target as background. The target hiding loss, based on the method described in [38], is formulated as follows:

(16) $$L_{hide}=-\frac{1}{n}\sum_{i=1}^{n}\left[{\hat{C}}_{i}^{p}\log\left(C_{i}^{p}\right)+\left(1-{\hat{C}}_{i}^{p}\right)\log\left(1-C_{i}^{p}\right)\right]$$

Here, *n* denotes the number of bounding boxes output by the target detector, and $C_{i}^{p}$ represents the confidence score of the detector for the *i*-th bounding box. ${\hat{C}}_{i}^{p}$ denotes the ground truth. To force the detector to ignore the targets in infrared images, we set ${\hat{C}}_{i}^{p}=0$. In this case,

(17) $$L_{hide}=-\frac{1}{n}\sum_{i=1}^{n}\log\left(1-C_{i}^{p}\right)$$

For the two-stage Faster R-CNN infrared target recognition model, during the generation of proposed regions, the probability distribution of all *N* proposed regions is given by $\left\{p_{1},p_{2},\ldots ,p_{n}\right\}$. To hide the target of interest in the infrared image, we design a foreground suppression strategy. This strategy reduces the foreground probability of the proposed regions in Faster R-CNN, causing the model to misclassify regions that originally belong to the target as background, thereby achieving target concealment. In this case, the target hiding loss for the Faster R-CNN infrared target recognition model is defined as

(18) $$L_{hide}=\frac{1}{N}\sum_{i=1}^{n}\max\left(0,f_{i}^{object}\left(x^{adv}\right)\right)$$

In this case, the object class confidence value corresponding to the *i*-th proposed region is $f_{\mathrm{object},i}\left(\cdot \right)$, and $f_{\mathrm{object},i}\left(\cdot \right)=p_{i}$. $x_{\mathrm{adv}}$ represents the adversarial patch applied to the original infrared image. The smaller the $L_{hide}$, the more likely it is that Faster R-CNN will classify the region as background. As a result, the detector assigns a lower confidence to the target region, making it more difficult for the model to recognize the target in the infrared image.

In infrared images, adversarial patches in the form of noise points often lack naturalness, making them difficult to deploy physically. To address this issue, a total variation (TV) smoothing loss term is introduced to constrain the local variations in the texture of the adversarial patch, thereby reducing the occurrence of isolated noise points. The loss is defined as follows:

(19) $$L_{\mathrm{smooth}}=\frac{1}{2HW}\sum_{i,j}\sqrt{{\left(p_{i+1,j}-p_{i,j}\right)}^{2}+{\left(p_{i,j+1}-p_{i,j}\right)}^{2}}$$

where *H* and *W* denote the height and width of the patch, respectively. The normalization factor 2HW accounts for the total number of horizontal and vertical gradient terms. *i* and *j* represent the pixel indices in the patch. This formula computes the gradient magnitude of adjacent pixels in the horizontal and vertical directions and suppresses abrupt pattern transitions by minimizing the total variation. Smooth gradient constraints play a critical role in physical-world robustness. In practical infrared patch printing or laser etching applications, isolated noise points are difficult to reproduce accurately due to material limitations, susceptibility to environmental degradation, and printing precision constraints. Incorporating this smoothing loss term ensures that the generated adversarial patches are more natural and continuous, with significantly reduced noise point artifacts.

We also introduce the structural similarity loss (SSIM), which helps ensure that the adversarial patch aligns more closely with the features of the infrared image while maintaining a minimal difference between the original and adversarial samples. The standard Structural Similarity Index (SSIM) [39] is formulated as

(20) $$SSIM\left(x,y\right)=\frac{\left(2\mu_{x}\mu_{y}+C_{1}\right)\left(2\sigma_{xy}+C_{2}\right)}{\left(\mu_{x}^{2}+\mu_{y}^{2}+C_{1}\right)\left(\sigma_{x}^{2}+\sigma_{y}^{2}+C_{2}\right)}$$

where the first term $\left(2\mu_{x}\mu_{y}+C_{1}\right)/\left(\mu_{x}^{2}+\mu_{y}^{2}+C_{1}\right)$ is used to evaluate the luminance similarity, the second term $\left(2\sigma_{xy}+C_{2}\right)/\left(\sigma_{x}^{2}+\sigma_{y}^{2}+C_{2}\right)$ is used to evaluate the contrast and structural similarity, and $\sigma_{xy}$ is the covariance between infrared images *x* and *y*. $\mu_{x}$, $\mu_{y}$, $\sigma_{x}$, and $\sigma_{y}$ are the mean and standard deviation of infrared images *x* and *y*, and $C_{1}$ and $C_{2}$ are constants. The SSIM value is always less than or equal to 1. SSIM calculates the local structural similarity by sliding an N×N window over the image and taking the mean of these local similarities as the overall similarity measure. A value closer to 1 indicates that the two infrared images are more similar.

When evaluating image similarity, the Structural Similarity Index (SSIM) simultaneously considers luminance, contrast, and structural information, aligning more closely with the human visual system’s perception mechanism. This task emphasizes the consistency of contrast and structure between the adversarial sample and the original infrared image. Given that infrared images often have lower resolution and the local adversarial patches have minimal impact on the overall brightness, we omit the luminance term (i.e., the first term) in Equation (20), which is based on the average gray levels $\mu_{x}$ and $\mu_{y}$, from the SSIM loss. Only the contrast and structural terms (i.e., the second term) are retained to improve the training convergence of the neural network model. The simplified SSIM formula is expressed as

(21) $$SSIM^{\prime }\left(x,y\right)=\frac{2\sigma_{xy}+C_{2}}{\sigma_{x}^{2}+\sigma_{y}^{2}+C_{2}}$$

Furthermore, we focus more on the structural similarity between the adversarial sample and the target. Since multiple targets may interact with each other, we do not compute the overall structural similarity of the image. Instead, we calculate the local structural similarity within each target detection box. For the *i*-th target identification box, let its corresponding original infrared image local region be $x_{i}$ and the adversarial patch local region be $y_{i}$. Then the structural similarity of this region is given by

(22) $$SSIM^{\prime }\left(x_{i},y_{i}\right)=\frac{2\sigma_{x_{i}y_{i}}+C_{2}}{\sigma_{x_{i}}^{2}+\sigma_{y_{i}}^{2}+C_{2}}$$

where $\sigma_{x_{i}}$ and $\sigma_{y_{i}}$ denote the standard deviations of the local regions $x_{i}$ and $y_{i}$, respectively, and $\sigma_{x_{i}y_{i}}$ represents the covariance between them. The constant is typically set to $c=9.4\times 10^{-4}$, with the variance denominator set to zero. Considering that different target frames contribute differently to the overall detection task, we weight the frame area by introducing a weight factor $\omega_{i}$, where $\omega_{i}=A_{i}/\sum_{j=1}^{n}A_{j}$ and $A_{i}$ is the area of the *i*-th target frame, satisfying the normalization constraint $\sum_{i=1}^{n}\omega_{i}=1$. By incorporating a weighted averaging approach, each target frame’s local structural similarity is aggregated. Since the SSIM metric indicates higher image similarity with larger values and the loss function requires minimization, we introduce a negative sign to transform the optimization objective into a minimization problem. The final SSIM loss function is defined as

(23) $$L_{SSIM}\left(x,y\right)=-\sum_{i=0}^{n}\omega_{i}\left(\frac{2\sigma_{x_{i}y_{i}}+C}{\sigma_{x_{i}}^{2}+\sigma_{y_{i}}^{2}+C}\right)$$

Here, *y* denotes the adversarial sample, and *x* represents the original infrared sample. *n* is the number of target bounding boxes in the image. A smaller value of $L_{\mathrm{SSIM}}\left(x,y\right)$ indicates that the adversarial sample preserves more target information from the original infrared image.

**Figure 4:**
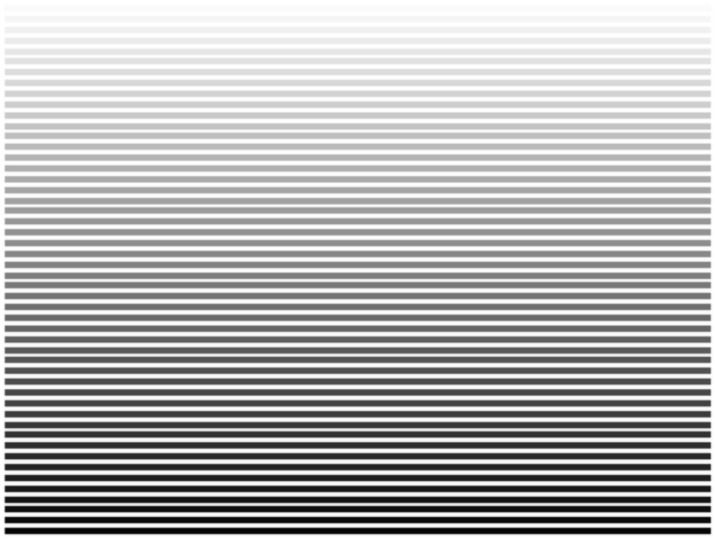
Visualization of the pixel value set.

To ensure that the generated adversarial patches are physically realizable in the real world, it is necessary to consider the infrared radiation characteristics of actual materials. The infrared radiation intensity that real materials can produce under specific environmental conditions is limited and cannot cover the full grayscale range of 0–255. Therefore, in this work, we randomly select 50 pixel values from a large number of real infrared images to construct a physically realizable pixel value set $S_{pixel}$, which is used to constrain the pixel value distribution of the adversarial patches. Figure 4 shows the visualization of these pixel values.

The adversarial patch pixel value loss in our algorithm is defined as

(24) $$L_{pixel}=\frac{1}{|S_{patch}|}\sum_{p_{j}\in S_{patch}}\min_{p\in S_{pixel}}\left|p_{j}-p\right|$$

where $p_{j}$ represents the pixel value of the *j*-th pixel in the adversarial patch, $S_{patch}$ denotes the set of pixel values in the adversarial patch, $S_{pixel}$ is the preset realizable pixel value set, and *p* represents a pixel value in $S_{pixel}$. This loss function constrains the patch pixel values to approach physically realizable infrared radiation intensities by calculating the distance between each patch pixel value and its nearest pixel value in the set $S_{pixel}$ and then averaging over all patch pixels. This constitutes a discrete set-matching constraint. A smaller $L_{c}$ indicates that the pixel values in the adversarial patch are closer to the preset set $S_{pixel}$, thus better conforming to the infrared radiation characteristics of actual materials. The physical meaning of this loss is as follows: the average deviation of each pixel in the patch from the realizable pixel set. In practical applications, the realizable pixel value set $S_{pixel}$ can be adjusted according to the infrared radiation intensity range of the materials.

#### 3.4.3. Magnitude-Level Analysis of Loss Terms

In the typical adversarial patch optimization process, different loss terms exhibit distinct numerical magnitude characteristics. Based on the experimental settings in this work, we performed a magnitude-level statistical analysis of each loss term to provide quantitative justification for the constraints in Equation (15).

The target hiding loss $L_{hide}$ is determined by the confidence output of the target detector. For YOLO applied to human targets, the confidence score $C_{i}^{p}$ generally falls within the range [0.7,0.95], corresponding to a single-target loss of [1.2,3.0]. In a typical multi-target scenario (n=2), according to Equation (10), the average single-target loss is approximately 1.9. After normalization, the typical value of $L_{hide}$ becomes 1.9/2=0.95, with a magnitude level of $O(10^{0})$. Considering variations in the number of targets and confidence values across different scenes, the numerical range of $L_{hide}$ is approximately [0.6,2.0].

The smooth loss $L_{smooth}$ is obtained by accumulating the gradient magnitudes of the patch pixels. According to Equation (19), the normalization factor is 2HW=2×300×300=180,000, and the total number of gradient terms is approximately (H−1)×W+H×(W−1)=179,400. For infrared images with medium texture quality, the typical pixel gradient magnitude lies within the range [5,40], with an average value of about 15. Therefore, the accumulated total gradient magnitude is 179,400×15=2,691,000. After dividing by the normalization factor, the typical value of $L_{smooth}$ is 2,691,000/180,000≈15, corresponding to a magnitude level of $O(10^{1})$. Considering variations in patch texture complexity, the numerical range of $L_{smooth}$ is approximately [8,30].

The value range of the SSIM loss $L_{SSIM}$ is constrained by the definition domain of the SSIM metric, i.e., [0,1]. According to Equation (22), in a multi-target scenario (n=2), the simplified SSIM values for each target box typically fall within [0.4,0.9]. Assuming that the areas of the two target boxes are approximately equal (with weights $\omega_{1}\approx \omega_{2}\approx 0.5$), and both SSIM values are 0.6, the weighted average becomes 0.5×0.6+0.5×0.6=0.6. Hence, the typical magnitude level of $L_{SSIM}$ is $O(10^{0})$. Considering variations in structural similarity at different optimization stages, the absolute value $|L_{SSIM}|$ generally falls within the range [0.4,0.9].

The pixel value loss $L_{pixel}$ is calculated by measuring the distance between the patch pixels and the predefined realizable pixel set. According to Equation (24), a patch contains $|S_{patch}|=300\times 300=90,000$ pixels, while the realizable pixel set size is $|S_{pixel}|=50$. When the pixel values of $S_{pixel}$ are uniformly distributed within the grayscale range [0,255], the average interval between adjacent realizable pixel values is 255/50≈5.1. For any given patch pixel $p_{i}$, the expected distance to its nearest realizable value is about 5.1/2=2.55. Thus, the total distance is approximately 90,000×2.55=229,500. After normalization by the patch size $|S_{patch}|=90,000$, the typical value of $L_{pixel}$ becomes 229,500/90,000=2.55, with a magnitude level of $O(10^{0})$. Considering that pixel intensities in real infrared images usually fall within [50,200], the actual mean distance is likely smaller, and the value range of $L_{pixel}$ is estimated to be within [1.5,3.5].

#### 3.4.4. Analysis of Weight Coefficients

To ensure that each loss term contributes reasonably to the overall loss and to avoid imbalance in magnitude, the target contribution of each weighted loss term is set within a comparable magnitude range. This design guarantees both gradient stability and training convergence.

Based on the magnitude-level analysis in Section 3.4.3, the representative numerical values of the loss terms are used as references for weight coefficient design. For $L_{hide}$, the median value within the range [0.6,2.0] is approximately 1.2; for $L_{smooth}$, and the typical value is 15; for $L_{SSIM}$, it is 0.6. For $L_{pixel}$, the representative value is 2.5.

To achieve magnitude-level balance, the target contribution of each weighted loss term is set to T=10. According to the principle of magnitude equilibrium, the initial weight ratios are calculated as follows:

(25) $$\begin{array}{cc} \lambda_{1}^{init} & =\frac{T}{L_{hide}}=\frac{10}{1.2}\approx 8.3,\;\lambda_{2}^{init}=\frac{T}{L_{smooth}}=\frac{10}{15}\approx 0.67\\ \lambda_{3}^{init} & =\frac{T}{L_{SSIM}}=\frac{10}{0.6}\approx 16.7,\;\lambda_{4}^{init}=\frac{T}{L_{pixel}}=\frac{10}{2.5}=4.0 \end{array}$$

However, if the weights are uniformly set such that each loss term contributes equally (e.g., $T_{i}=10$ for all terms), this fails to reflect the dominant role of target hiding as the primary objective. Therefore, we need to adjust the weights based on hierarchical balance. According to the target percentage allocations specified in Section 3.4.1 (target hiding: 60–70%, smoothness: 10–20%, SSIM: 5–10%, pixel constraints: 10–20%), we perform weight adjustment.

The loss term proportions are set as follows: target hiding 65%, smoothness 15%, SSIM 8%, pixel constraints 12%. Based on the magnitude estimation from Equation (25) (where each contribution averages to 10 and the total loss is approximately 4×10=40), we set L=40. According to the percentage constraints, we obtain

(26) $$\begin{array}{cc} \lambda_{1}\times 1.2 & =0.65\times 40=26,\;\lambda_{2}\times 15=0.15\times 40=6\\ \lambda_{3}\times 0.6 & =0.08\times 40=3.2,\;\lambda_{4}\times 2.5=0.12\times 40=4.8 \end{array}$$

Solving yields the weight configuration: $\left(\lambda_{1},\lambda_{2},\lambda_{3},\lambda_{4}\right)=\left(22,0.4,5,2\right)$. Under this configuration, the contribution of each weighted loss term is $\lambda_{1}\times L_{\mathrm{hide}}=22\times 1.2=26.4$ (65.0% proportion), $\lambda_{2}\times L_{\mathrm{smooth}}=0.4\times 15=6.0$ (15.0% proportion), $\lambda_{3}\times L_{\mathrm{SSIM}}=5\times 0.6=3.0$ (7.5% proportion), and $\lambda_{4}\times L_{\mathrm{pixel}}=2\times 2.5=5.0$ (12.5% proportion). The total loss is approximately $L_{\mathrm{total}}\approx 26.4+6.0+3.0+5.0=40.4$. Experimental validation is detailed in the sensitivity analysis in Section 4.3.3 (see Section 4.3.3 for sensitivity analysis).

### 3.5. Optimization Strategy

In this study, the adversarial patch is trained using a gradient-based optimization method. According to the total loss function L defined in Equation (15), the patch parameters are updated by minimizing L. To enhance the convergence sensitivity and stability of the optimization process, the Adam (Adaptive Moment Estimation) optimizer is employed for parameter updates.

For the *t*-th iteration, the gradient of the total loss function L with respect to the patch is first computed as

(27) $$g_{t}=\nabla_{\mathrm{patch}}L=\nabla_{\mathrm{patch}}\left(\lambda_{1}L_{hide}+\lambda_{2}L_{smooth}+\lambda_{3}L_{SSIM}+\lambda_{4}L_{c}\right)$$

Here, $\nabla_{\mathrm{patch}}$ denotes the gradient of the loss function with respect to the pixel values of the adversarial patch, reflecting how each pixel affects the loss. It determines the update direction that minimizes the overall loss function.

When the loss function $L_{\mathrm{hide}}$ penalizes the detector’s confidence scores, the gradients $\nabla_{\mathrm{patch}}L_{\mathrm{hide}}$ generated through backpropagation exhibit larger magnitudes at locations corresponding to discriminative features relied upon by the detector. This occurs because the detector learns to depend on these features during training (such as thermal radiation edges of human bodies, torso contours, and temperature contrast regions between targets and backgrounds) for classification, making its output more sensitive to pixel changes at these locations. Consequently, the spatial distribution of gradients directly reflects these critical positions. The Adam optimizer adaptively adjusts the update step size based on the gradients $g_{t}$ computed according to Equation (27), enabling pixel updates in the patch to automatically receive larger perturbation magnitudes at high-gradient locations. Since the patch is placed within the attention region identified by Grad-CAM, the optimization process spatially focuses on detector-sensitive areas while targeting discriminative feature pixels that the detector relies upon within that region. Therefore, the final generated patch perturbation automatically inherits the spatial distribution of the detector’s discriminative features through gradient backpropagation: perturbation magnitudes are larger at critical feature locations and smaller at non-critical positions.

Next, the first- and second-order moment estimates (i.e., the exponential moving averages of the gradient and its square) are updated as follows:

(28) $$m_{t}=\beta_{1}m_{t-1}+\left(1-\beta_{1}\right)g_{t}$$

(29) $$v_{t}=\beta_{2}v_{t-1}+\left(1-\beta_{2}\right)g_{t}^{2}$$

In these expressions, $\beta_{1}$ and $\beta_{2}$ represent the exponential decay rates for the first- and second-order moments, respectively. Since $m_{0}$ and $v_{0}$ are initialized to zero, a bias correction is required during the early training stage to compensate for initialization bias.

(30) $${\hat{m}}_{t}=\frac{m_{t}}{1-\beta_{1}^{t}}$$

(31) $${\hat{v}}_{t}=\frac{v_{t}}{1-\beta_{2}^{t}}$$

Finally, the update rule for the adversarial patch can be expressed as

(32) $${\mathrm{patch}}^{\left(t+1\right)}={\mathrm{patch}}^{\left(t\right)}-\alpha \frac{{\hat{m}}_{t}}{\sqrt{{\hat{v}}_{t}}+\varepsilon }$$

where α denotes the learning rate and ε is a small constant used to prevent division by zero (set to $10^{-8}$ in this study). In the experiments, the hyperparameters are set as $\beta_{1}=0.9$, $\beta_{2}=0.999$, and α=0.01.

To ensure that the generated adversarial patch remains within the valid pixel range, a clipping operation is applied after each update:

(33) $${\mathrm{patch}}^{\left(t+1\right)}=\mathrm{clip}\left({\mathrm{patch}}^{\left(t+1\right)},0,255\right)$$

This clipping operation guarantees that all pixel values of the patch are constrained within the valid grayscale range of [0,255], thereby maintaining consistency with the physical properties of infrared images.

### 3.6. Adversarial Patch Region Constraints and Robustness Enhancement

**Figure 5:**
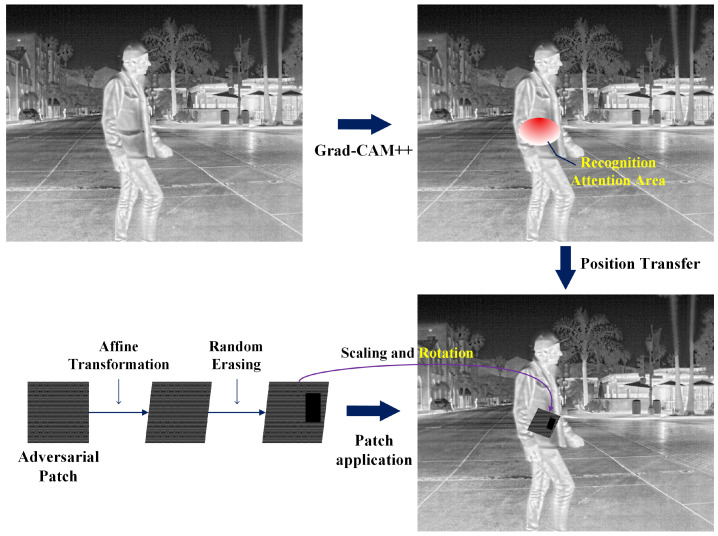
Adversarial patch region constraints and robustness enhancement.

For the generated adversarial patch, in order to achieve the optimal attack effect on the target recognition model, we constrain the patch to the most critical region that the model focuses on. To this end, we first employ the Grad-CAM algorithm [23] to locate this region and then apply the adversarial patch within it. In addition, considering that the motion of data acquisition platforms such as UAVs, or the movement of targets themselves, may cause variations in the imaging viewpoint and target size, we apply affine transformations and scaling operations to the adversarial patch. Furthermore, to enhance the generalization capability of the patch and prevent overfitting to a specific recognition model, we introduce the random erasing strategy [40], which randomly masks parts of the patch during training. This dynamically adjusts the patch shape, thereby increasing its diversity and improving its robustness under different background environments. Figure 5 illustrates this process.

In this work, the target of interest is human bodies; therefore, we only focus on human regions. As shown in the figure, the Recognition Attention Area can be visualized in the form of a heatmap using the Grad-CAM algorithm, which highlights the importance of each location in the input infrared image for the decision-making process of the object detector.

The Grad-CAM algorithm computes the gradient of the target class *c* with respect to the feature map $A^{k}$, generating a *Class Activation Map* (CAM). Specifically, for the *k*-th feature map, the importance weight $\alpha_{c}^{k}$ is calculated as

(34) $$\alpha_{c}^{k}=\frac{1}{Z}\sum_{i}\sum_{j}\frac{\partial y^{c}}{\partial A_{i,j}^{k}}$$

where $y^{c}$ denotes the score corresponding to the target class *c*, $A_{i,j}^{k}$ represents the activation value at the spatial position (i,j) of the *k*-th feature map, and *Z* is the normalization factor corresponding to the spatial size of the feature map. The class activation map $L_{Grad-CAM}^{c}$ is then obtained by combining all feature maps through a weighted summation:

(35) $$L_{Grad-CAM}^{c}=\mathrm{ReLU}\;\left(\sum_{k}\alpha_{c}^{k}A^{k}\right)$$

where the ReLU function ensures that only regions contributing positively to the target class are preserved. The resulting activation map is visualized as a heatmap, indicating the importance of each spatial location in the input infrared image for the classifier’s decision. Regions highlighted in red correspond to areas where the detector focuses more strongly on the target. To transform the activation map into a normalized attention map for subsequent region selection, $L_{Grad-CAM}^{c}$ is first normalized as follows:

(36) $$M\left(x,y\right)=\frac{L_{Grad-CAM}^{c}\left(x,y\right)-\min\left(L_{Grad-CAM}^{c}\right)}{\max\left(L_{Grad-CAM}^{c}\right)-\min\left(L_{Grad-CAM}^{c}\right)}$$

Then, a threshold τ (set to 0.7 in this study) is applied to extract the high-activation region Ω as the candidate area for patch placement:

(37) Ω={(x,y)∣M(x,y)≥τ}

Considering the feasibility of physical implementation, it is generally more reasonable to paste adversarial patches on the upper body rather than the lower body. Therefore, a position bias weighting function W(y) is introduced when sampling locations within region Ω, guiding the placement of the patch toward the upper part of the detected bounding box:

(38) $$y_{\Omega }=\frac{\sum_{(x,y)\in \Omega }y\cdot M\left(x,y\right)}{\sum_{(x,y)\in \Omega }M\left(x,y\right)}$$

Here, $y_{\Omega }$ denotes the weighted centroid coordinate of the activation region Ω. Then, according to the relative position of $y_{\Omega }$ within the detection bounding box, the parameter β is adaptively adjusted as

(39) $$\beta =\beta_{\min}+\left(\beta_{\max}-\beta_{\min}\right)\cdot \frac{y_{\Omega }-y_{\mathrm{top}}}{y_{\mathrm{bottom}}-y_{\mathrm{top}}}$$

In this equation, $y_{\mathrm{top}}$ and $y_{\mathrm{bottom}}$ denote the top and bottom coordinates of the detection bounding box, respectively. $\beta_{\min}$ and $\beta_{\max}$ represent the lower and upper bounds of the positional bias strength, which are set to $\beta_{\min}=0.3$ and $\beta_{\max}=2.0$ in this study. This adaptive strategy ensures that when the activation region Ω lies in the upper part of the detection box, β becomes smaller, allowing the patch to be guided mainly by the activation intensity. Conversely, when Ω is located in the lower part of the detection box, β increases, strengthening the upward bias toward the upper body. Based on the adjusted β value, the positional weighting function W(y) is defined as

(40) $$W\left(y\right)=\exp\;\left(-\beta \frac{y-y_{\mathrm{top}}}{y_{\mathrm{bottom}}-y_{\mathrm{top}}}\right)$$

By combining the activation intensity and the positional bias, we define a joint sampling probability distribution P(x,y) as

(41) $$P\left(x,y\right)=\frac{M(x,y)\cdot W(y)}{\sum_{(x^{\prime },y^{\prime })\in \Omega }M\left(x^{\prime },y^{\prime }\right)\cdot W\left(y^{\prime }\right)},\;\left(x,y\right)\in \Omega$$

During training, in each iteration, the patch center is randomly sampled from region Ω according to the probability distribution P(x,y), denoted as $\left(x_{p},y_{p}\right)$. This strategy introduces a positional prior that favors the upper body while retaining randomness, which ensures that (1) the model does not overfit to a fixed region, (2) the diversity of training samples is improved, and (3) the patch placement remains physically plausible and well aligned with the human upper-body area in real-world scenarios. The size of the patch is also adapted according to the size of the target bounding box.

To improve the robustness of the attack, three aspects are considered: (1) The distance between the infrared camera and the pedestrian target may vary, leading to different apparent scales of the patch. (2) Due to differences in camera viewpoints, the visual appearance of the patch can differ. (3) Infrared images captured at different distances exhibit scale variations in the patch relative to the target. To address these issues, a series of geometric transformations are applied to the patch during training. The transformation can be expressed as

(42) T(x)=A(x)+b

where *x* denotes the original coordinates of a pixel within the adversarial patch, T(x) represents the transformed coordinates, *A* is a 2×2 affine transformation matrix, and *b* is a translation vector. During training, the transformation parameters are randomly sampled: the rotation angle is drawn uniformly from $\left[0^{\circ },30^{\circ }\right]$, which enhances the patch’s invariance to changes in viewing angle and scale. After training, the rectangular patch that produces the best attack effect is fixed as the final adversarial patch.

**Figure 6:**
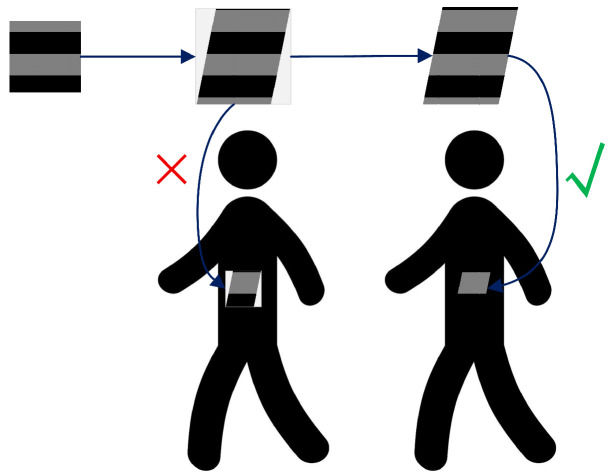
Cropping of adversarial patch after affine transformation.

To determine the optimal vertical placement within the bounding box, the Grad-CAM heatmap is used to estimate the vertical distribution of attention. Let $y_{\mathrm{cam}}$ denote the vertical coordinate of the CAM activation centroid, and let $w_{\mathrm{cam}}$ represent the corresponding activation intensity weight. The patch loss is then defined as

(43) $$l_{\mathrm{patch}}=\beta \cdot w_{\mathrm{cam}}\;\beta \in \left[0.4,0.6\right]$$

where β controls the relative importance between the target region and the background region. A larger β places more emphasis on the target region, improving attack effectiveness but potentially increasing detectability or causing unrealistic placement. A smaller β reduces physical attack strength. Therefore, β is set within [0.4,0.6] to balance realism and effectiveness. In practice, the final adversarial patch is sampled randomly within the region indicated by the activation distribution and positional bias. This randomization enhances the patch’s robustness to different human postures and detection scenarios. During deployment, only the valid rectangular area of the trained patch is retained, while any excess outside this area is cropped to remove background regions, as illustrated in Figure 6.

Furthermore, to enhance the generalization ability of the adversarial patch, we introduce a local masking strategy (random erasing) during the training phase. The core idea of this strategy is to randomly mask a portion of the adversarial patch during each training iteration, thereby preventing the model from overfitting to specific local features of the patch.

The random erasing operation is formally defined as follows. Given a patch of size W×H, in each training iteration, a random erasing operation is performed with a probability of 0.6. The width ratio $r_{w}$ and height ratio $r_{h}$ of the erased region are independently sampled as

(44) $$r_{w}∼U\left(0.1,0.3\right),\;r_{h}∼U\left(0.1,0.3\right)$$

where U(·) denotes a uniform distribution. The width $w_{e}$ and height $h_{e}$ of the erased region are then calculated as

(45) $$w_{e}=r_{w}\cdot W,\;h_{e}=r_{h}\cdot H$$

The coordinates of the top-left corner $\left(x_{e},y_{e}\right)$ of the erased region are uniformly sampled within the following range:

(46) $$x_{e}∼U\left(0,W-w_{e}\right),\;y_{e}∼U\left(0,H-h_{e}\right)$$

The pixels within the erased region are filled with the mean pixel value of the patch. Through this random erasing operation, a portion of the patch is occluded differently in each training iteration, which effectively prevents the adversarial patch from overfitting to the recognition model or the training data, thereby improving its robustness to various targets.

During deployment, only affine transformations such as rotation and scaling are applied to the generated patch to match the real-world viewing angles and distances, while maintaining its physical feasibility for printing and placement.

## 4. Experiments

### 4.1. Datasets and Hardware Configuration

**Figure 7:**
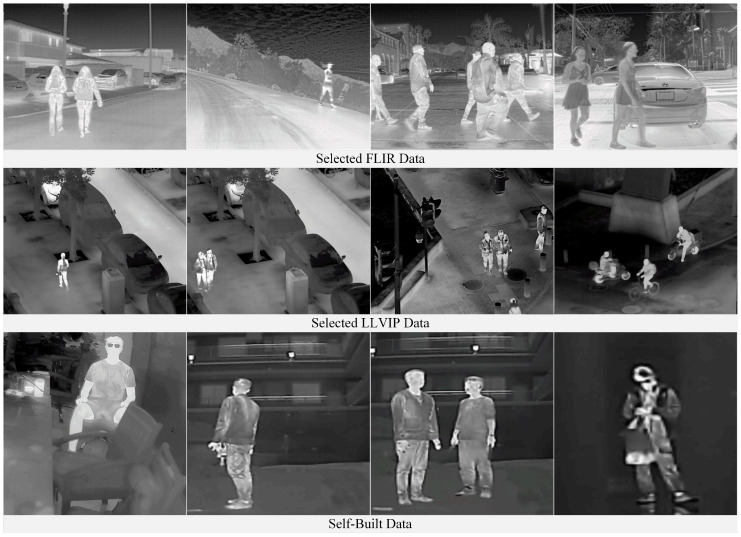
Constructed infrared image datasets.

The datasets used in this study encompass diverse perspectives and scene characteristics to enhance the generalization ability of the adversarial patch. The FLIR dataset mainly consists of ground-level views, captured in outdoor scenes such as streets and highways, covering both daytime and nighttime conditions. This dataset reflects the complex natural environments commonly encountered in ground-based surveillance systems. In contrast, the LLVIP dataset adopts a top-down perspective, with wide-area scenes such as urban roads, squares, and parking lots, which better resemble the observational perspectives of UAV. Since this study focuses exclusively on humans as the target class, we applied the following filtering criteria to both datasets: (1) the infrared image must contain the target class “person”; (2) the body height of the person in the infrared image must be at least 100 pixels. After filtering, we obtained 2000 ground-view infrared images from the FLIR dataset and 2000 top-view infrared images from the LLVIP dataset. Considering that the above datasets primarily contain outdoor scenes, we additionally collected 400 infrared images in indoor environments to expand the applicability of the generated adversarial patches to broader scenarios. These images were captured in typical indoor settings such as offices, corridors, and lobbies. Figure 7 shows representative samples of the constructed dataset.

In total, we constructed a dataset comprising 4400 infrared images, where 3520 images were used for training and 880 images for testing. The hardware environment consisted of an NVIDIA GeForce RTX 3090 GPU with 24 GB of memory, an Intel(R) Xeon(R) CPU at 2.50 GHz, and 96 GB of RAM, running on Ubuntu 20.04.

### 4.2. Evaluation Metrics for Adversarial Attack Effectiveness

The goal of this experiment is to make the infrared target recognition model fail to detect humans. To evaluate the effectiveness of the adversarial attack from multiple perspectives, we use the Average Precision (AP), Attack Success Rate (ASR), and the change in AP (ΔAP) as comprehensive metrics.

The Average Precision (AP) is a standard metric used to evaluate detection performance within the target region. It is defined as the area under the precision–recall curve and can be calculated as

(47) $$AP=\int_{0}^{1}P\left(R\right)\;dR=\int_{0}^{1}\frac{TP(R)}{TP(R)+FP(R)}\;dR,\;R=\frac{TP}{TP+FN}$$

where TP denotes the number of true positives, FP represents false positives, and FN indicates false negatives. A lower AP value implies that the detector is more vulnerable to adversarial attacks and tends to misclassify the target.

The Attack Success Rate (ASR) is defined as the ratio of missed detections caused by the adversarial attack to the number of correctly detected targets under the baseline condition, and it is calculated as

(48) $$ASR=\frac{N_{\mathrm{miss}}}{N_{\mathrm{trials}}}=\frac{N_{\mathrm{trials}}-N_{\mathrm{att}}}{N_{\mathrm{trials}}}$$

where $N_{\mathrm{trials}}$ denotes the number of targets detected by the model without attack (i.e., baseline performance), and $N_{\mathrm{att}}$ represents the number of targets successfully detected after the adversarial attack. Therefore, $N_{\mathrm{miss}}=N_{\mathrm{trials}}-N_{\mathrm{att}}$ indicates the number of missed detections resulting from the attack. A higher ASR indicates a better performance of the adversarial attack algorithm, meaning the target detector is less likely to detect the target. The change in Average Precision (ΔAP) is calculated as the absolute difference between the AP value before the attack and after the attack:

(49) $$\Delta AP=\left|AP_{pre}-AP_{last}\right|$$

This ΔAP metric is useful for comparing the effectiveness of different adversarial attack algorithms applied to the same object detector. A larger ΔAP indicates a more effective attack, as the adversarial attack algorithm causes a greater drop in AP value for the specific object detector.

### 4.3. Target Recognition Model and Adversarial Patch Training

#### 4.3.1. Target Recognition Model Initialization Training

**Figure 8:**
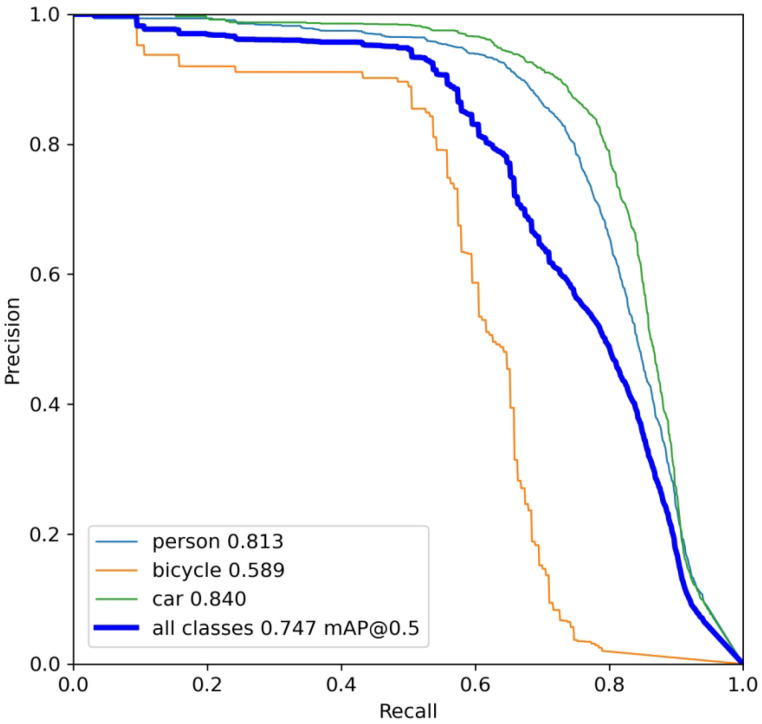
Precision–recall (P-R) curve of YOLOv5s detector.

Adversarial patches do not impose specific requirements on the attacked target recognition algorithm. In this study, we prioritize using YOLOv5s due to its relatively fewer parameters, faster inference speed, and ease of deployment on embedded systems. Based on the dataset constructed in this work, the recognition model was pre-trained for 200 epochs. As shown in Figure 8, the YOLOv5s object detector demonstrated satisfactory Average Precision performance, with the initial AP for the “person” category reaching 81.3%.

#### 4.3.2. Attention Heatmaps of the Target Recognition Model

**Figure 9:**
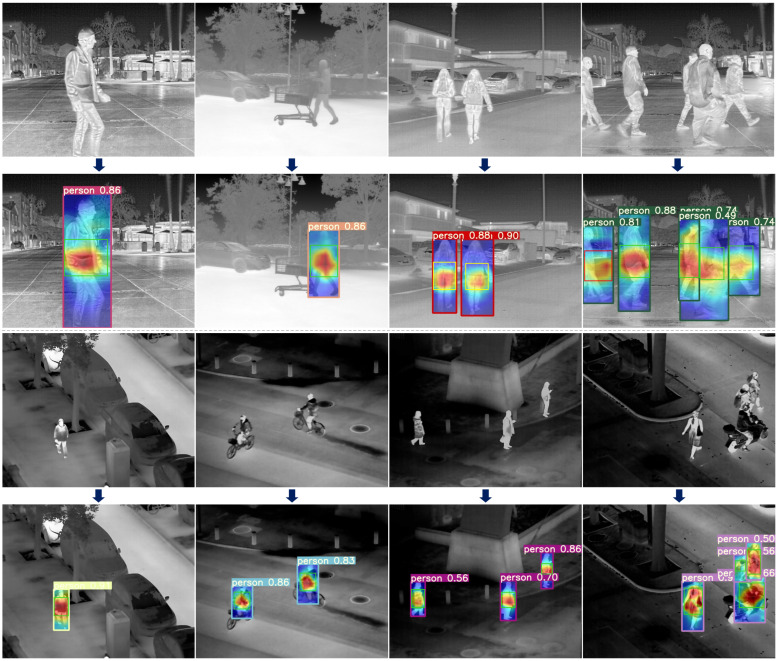
Attention heatmaps of YOLOv5s using Grad-CAM++.

We utilize YOLOv5s in combination with the Grad-CAM++ algorithm to visualize the salient feature regions in the target image. These regions have a stronger influence on the model’s decision-making process, and any perturbations in these regions are likely to significantly degrade model performance, thereby enabling more efficient adversarial attacks. The object detector is configured with a confidence threshold of 0.25 and an IoU threshold of 0.5, preventing multiple redundant bounding boxes for a single target. To ensure the representativeness of the results, these thresholds align with those commonly used in industrial applications. Figure 9 shows the rendered attention heatmaps of YOLOv5s.

Since this study mainly focuses on attacking the “person” category, we aim to apply the adversarial patch within the detection bounding boxes of humans. The exact location inside the bounding box is determined using Grad-CAM++. In the heatmaps, the redder the area, the more attention the object detector places on that region. The adversarial patch is pasted near these red areas, with random variation allowed within the region.

As shown in the figure, we mark approximate random regions with green, yellow, and red bounding boxes. Green boxes indicate normal regions, while red boxes correspond to areas with slight deviations, possibly caused by partial occlusion of the human body (e.g., the first person in the upper-right corner). The yellow box (third image in the top row) represents an attention region biased towards the lower part of the detection box, suggesting it may correspond to the lower limbs. However, considering practical constraints in physical-world implementation, we prefer the adversarial patch to appear on the upper body. Therefore, in such cases, we slightly shift the patch upwards. For targets with larger attention regions (e.g., the first person in the bottom-right corner), we increase the probability of placing the adversarial patch in the upper body region during random placement.

To ensure that the adversarial patch achieves a balance among attack effectiveness, visual naturalness, and physical realizability, it is necessary to determine a reasonable set of loss function weights. In the next section, a sensitivity analysis is conducted to determine the optimal weight configuration.

#### 4.3.3. Sensitivity Analysis of Loss Weight Coefficients

To verify the rationality of the empirically derived weight configuration $\lambda^{*}=\left(22,0.4,5,2\right)$ described in Section 3.4.4, a perturbation test is performed by varying each weight within ±50% of its nominal value. In each experiment, only one weight coefficient is perturbed while the others remain constant. The Attack Success Rate (ASR), structural similarity (SSIM), and each loss component value are evaluated on a dataset of 600 infrared images to analyze the characteristics of different configurations.

The theoretical optimal weights $\lambda^{*}=\left(\lambda_{1}^{*},\lambda_{2}^{*},\lambda_{3}^{*},\lambda_{4}^{*}\right)=\left(22,0.4,5,2\right)$ are used as the baseline. For the sensitivity test, each weight coefficient is multiplied by a scaling factor selected from the set {0.5,0.8,1.0,1.2,1.5}, corresponding to a perturbation range of ±50%. Each configuration is tested under identical experimental conditions, and all parameters are trained until convergence.

**Figure 10:**
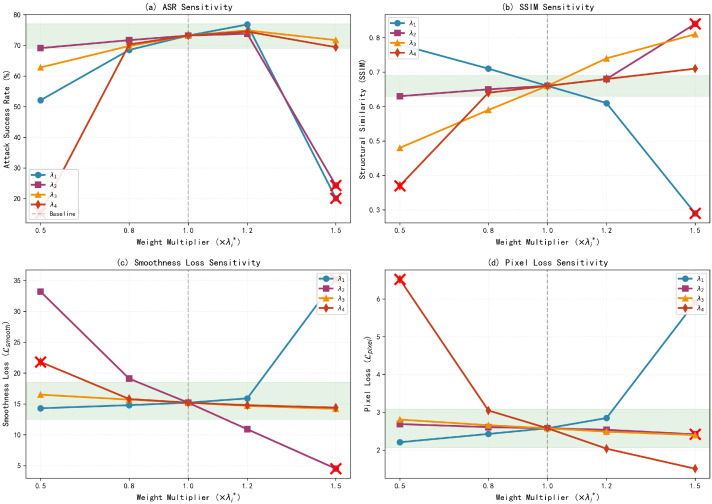
Weight Sensitivity Analysis. The red multiplication signs (×) indicate cases where the model fails to converge.

Table 2 shows the system performance under different scaling factors of key weight parameters. The results indicate that variations in weight coefficients cause noticeable differences in performance metrics, revealing significant sensitivity patterns among different loss weights. Figure 10 illustrates the corresponding trend curves of weight influence.

From the perspective of attack effectiveness (a), $\lambda_{1}$ and $\lambda_{4}$ have the most significant impact on the Attack Success Rate (ASR). When $\lambda_{1}$ increases to 1.2×, the ASR reaches its peak at 76.8%, but further increases cause a sharp decline. Conversely, reducing $\lambda_{4}$ to 0.5× leads to a drastic drop in ASR to 15.2%, indicating that these two weights directly control the balance between adversarial attack intensity and pixel constraints. Regarding visual quality (b), $\lambda_{2}$ shows a strong negative correlation with SSIM. Although an excessive smoothing constraint increases SSIM, it sacrifices attack performance. On the other hand, an overly large $\lambda_{1}$ results in structural distortion (SSIM = 0.29).

In terms of loss term stability (c–d), $\lambda_{2}$ predominantly controls spatial smoothness (with the steepest $L_{\mathrm{smooth}}$ slope), while $\lambda_{4}$ linearly regulates pixel distribution legality (with a correlation coefficient of −0.96 with $L_{\mathrm{pixel}}$). The convergence boundaries identified by the experiments are $\lambda_{1}\in \left[11,24.6\right]$, $\lambda_{2}\leq 0.6$, $\lambda_{3}\geq 2.5$, and $\lambda_{4}\geq 2.4$, revealing the constraint space for multi-objective optimization. Three non-convergent points (marked by X) demonstrate that exceeding these boundaries triggers training failure. Within a ±20% perturbation range, all metrics remain stable (green region), validating the robustness of the baseline configuration.

Based on the comprehensive evaluation above, we adopt the theoretically derived weight configuration $\lambda^{*}=\left(22,0.4,5,2\right)$ as the final loss function weight allocation. This configuration achieves the optimal balance across four dimensions: attack performance (ASR = 73.2%), physical feasibility $L_{\mathrm{pixel}}$ = 2.58), visual naturalness (SSIM = 0.66), and parameter stability. Furthermore, all weights are positioned at the center of the experimentally validated convergence region, ensuring reliability in practical applications.

#### 4.3.4. YOLOv5s + GADP Algorithm Adversarial Patch Training Process

**Figure 11:**
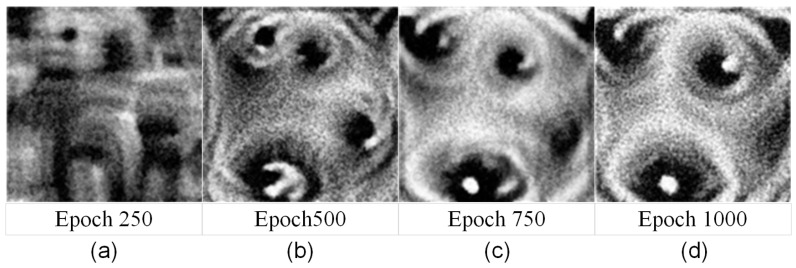
Infrared adversarial patches generated by the GADP algorithm.

The optimal weight configuration determined in Section 4.3.3, (10,0.05,2,0.1). For the YOLOv5s object detector, adversarial patches generated using the GADP algorithm constructed in this study are shown in Figure 11. Panels (a), (b), (c), and (d) represent adversarial patch images at training steps 250, 500, 750, and 1000, respectively.

**Figure 12:**
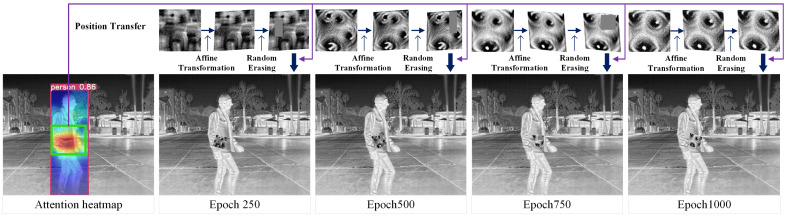
Visualization of the infrared adversarial patch training process.

Additionally, during the training process, we performed affine transformations, size adjustments, and random erasing to enhance the robustness of the adversarial patches. When constraining the location of the adversarial patch, we combined the attention heatmap and randomly located and rotated the patch within the green bounding box in the figure. The specific training process visualization is shown in Figure 12.

**Figure 13:**
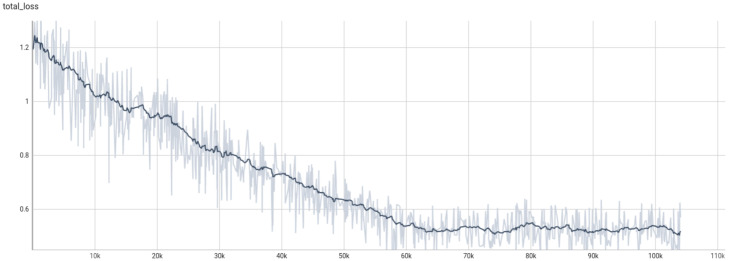
Loss function value variation during GADP algorithm training.

The loss function variation during the GADP algorithm’s adversarial patch training process is shown in Figure 13.

In the experiments, training was conducted for 1000 epochs (approximately 104,000 iterations). The horizontal axis represents the number of iterations, and the vertical axis denotes the loss function value. The light-colored curve in Figure 13 shows the instantaneous loss at each iteration, and the dark-colored curve shows the moving-averaged loss (with a window size of 1000). As observed from Figure 13, during the first 60,000 iterations, the moving-averaged loss decreases from an initial value of about 1.25 to around 0.5. Thereafter, the loss enters a plateau and remains stable until the end of training. To evaluate if the training satisfies the convergence condition, a post hoc analysis was performed based on the statistical stability of the loss. Specifically, the later stage of training (from 60 k to 104 k iterations) was divided into four segments, and the coefficient of variation (the ratio of standard deviation to mean) of the average loss in each segment was calculated. When the coefficient of variation is below 5%, the loss is considered stable, indicating convergence. Statistical results show the average losses of the four segments are approximately 0.51, 0.48, 0.52, and 0.50, with a standard deviation of about 0.016 and a mean of about 0.50, yielding a coefficient of variation of roughly 3.2%, which satisfies the convergence criterion. Due to the adversarial nature of optimization, the loss function in adversarial training typically oscillates around an equilibrium point after convergence. The above results show the loss has stabilized around 0.5, confirming that the training has reached convergence.

#### 4.3.5. Adversarial Patch Generation for Other Detection Algorithms Using GADP

**Figure 14:**
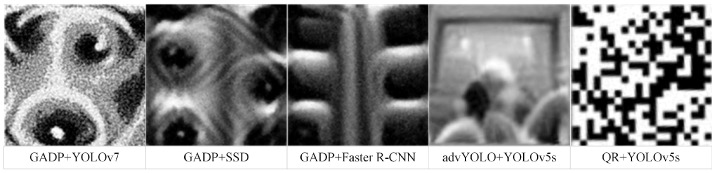
Adversarial patches generated by combining the GADP algorithm with other target recognition algorithms.

This study also generates adversarial patches using the GADP algorithm combined with several other classic target recognition algorithms, as shown in Figure 14. The figure presents adversarial patches generated by combining GADP with YOLOv7 [41], SSD [42], and Faster R-CNN [43], as well as adversarial patches generated by combining QR Attack [36] and advYOLO [35] with the YOLOv5s object detector. The QR Attack method designs adversarial QR code patches to attack infrared target detectors. The advYOLO Patch algorithm targets the “person” class, creating an adversarial patch that prevents the infrared target recognition model from detecting humans. Notably, the adversarial patches generated by GADP + YOLOv7 are quite similar to those generated by GADP + YOLOv5s, which can be attributed to the high similarity between the YOLOv7 and YOLOv5s models.

#### 4.3.6. Complexity Analysis of the GADP Training Algorithm

The experimental environment utilized an NVIDIA RTX 3090 GPU with 24 GB memory. The training dataset consisted of 3520 infrared images with a resolution of 640 × 640 pixels, and the batch size was set to 8. The adversarial patch template had a size of 300 × 300 pixels.

Time complexity: Each training iteration consists of five stages: (1) forward inference on 640 × 640 input images using the YOLOv5s model, which contains about 7.3 million parameters; (2) patch application and data augmentation, involving affine transformations and random erasing on 32 images; (3) forward propagation with Bernoulli Stochastic Dropout (BSD) applied to the CSP modules in the backbone network; (4) loss computation over about 25,200 predicted bounding boxes; and (5) backpropagation and patch update, optimizing only 270,000 patch parameters. Since the computational cost of forward and backward propagation dominates other operations, the major computation per iteration comes from model inference (about 7.3 M parameters) and batch processing (32 images). The training process comprises 104,000 iterations, with an estimated total computational load of $7.6\times 10^{14}$ floating-point operations. The measured time per iteration is about 0.16 s, resulting in a total training duration of around 4.6 h.

Space complexity: The overall memory consumption mainly consists of (1) 32 input images (640 × 640) and patch data, occupying about 190 MB; (2) intermediate feature maps generated by the backbone, neck, and head networks, consuming about 2.5 GB; (3) BSD Bernoulli sampling data, accounting for around 200 MB; and (4) gradient and optimizer states for the 270,000 optimized patch parameters, requiring about 10 MB. The observed peak GPU memory usage was about 5.2 GB and remained stable throughout training, with feature maps accounting for 70% of the total. Compared with end-to-end detector training (which requires optimizing 7.3 M parameters), the GADP algorithm updates only 270 K patch parameters, reducing memory overhead for gradients and optimizers by about 96%. This reduction highlights the practicality of GADP in resource-constrained environments.

### 4.4. Adversarial Patch Application Results

**Figure 15:**
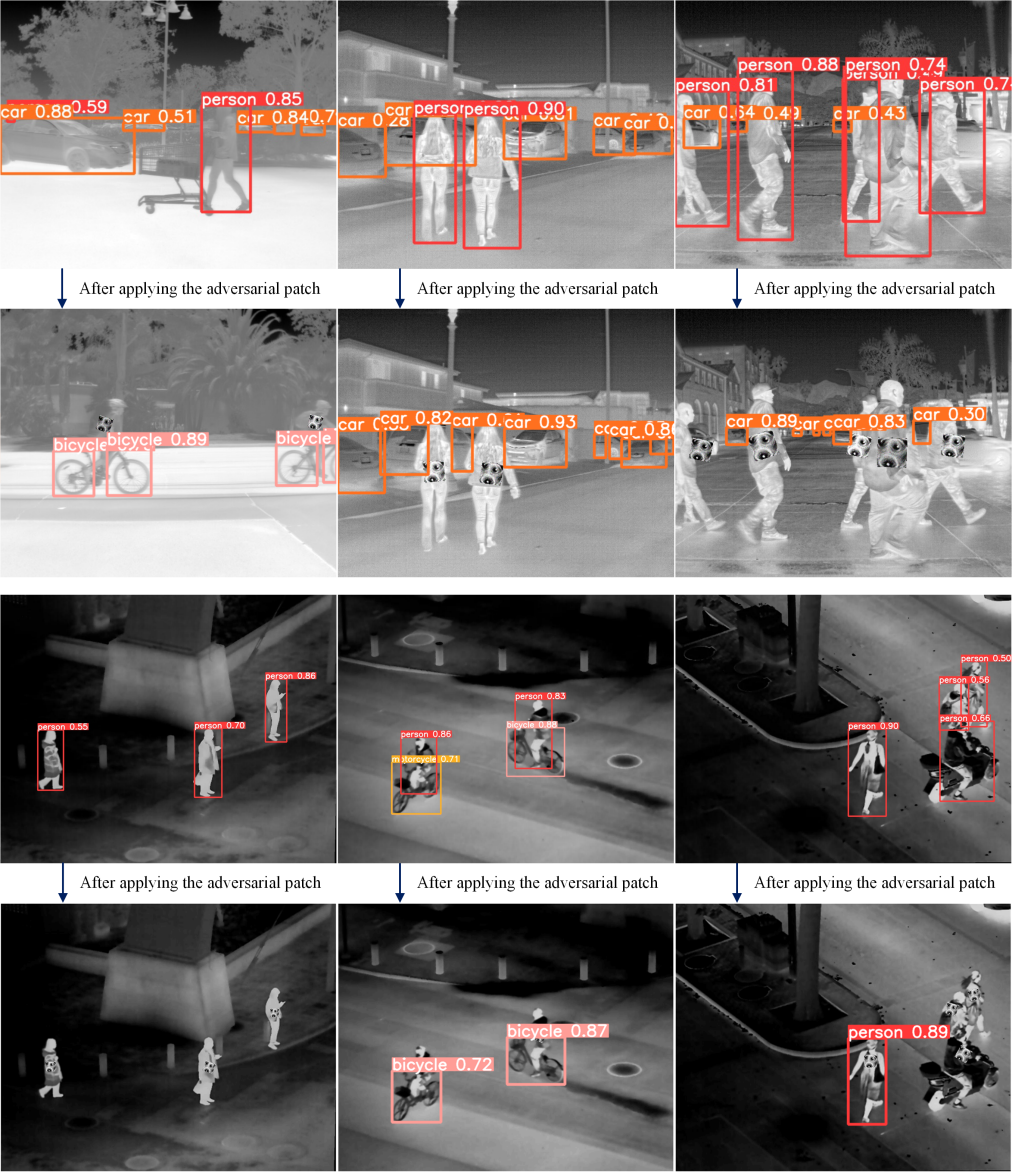
Detection results for the original and adversarial sample images. Colored bounding boxes represent different detected object classes: deep red for person, orange for car, light red for bicycle, and yellow for motorcycle.

Figure 15 presents the YOLOv5s detection results for the infrared image and the adversarial sample image. The scenarios include single and multiple persons, with backgrounds such as roads, forests, buildings, and streets. In the infrared image, YOLOv5s can correctly detect categories such as “person,” “car,” and “bicycle.” However, after applying the adversarial patch to generate the adversarial sample, YOLOv5s fails to detect the “person” in the adversarial sample, though it can still detect other categories. The infrared recognition model struggles more with feature representation in complex backgrounds, and the interference from the adversarial patch further diminishes the model’s ability to extract features, leading to detection failure. An example of a failed attack (first person in the lower-right corner) is shown in the figure. This may be due to the large attention region (as seen in Figure 9 in the lower-right corner), with the small size of the adversarial patch insufficient to disrupt the model’s attention effectively.

**Figure 16:**
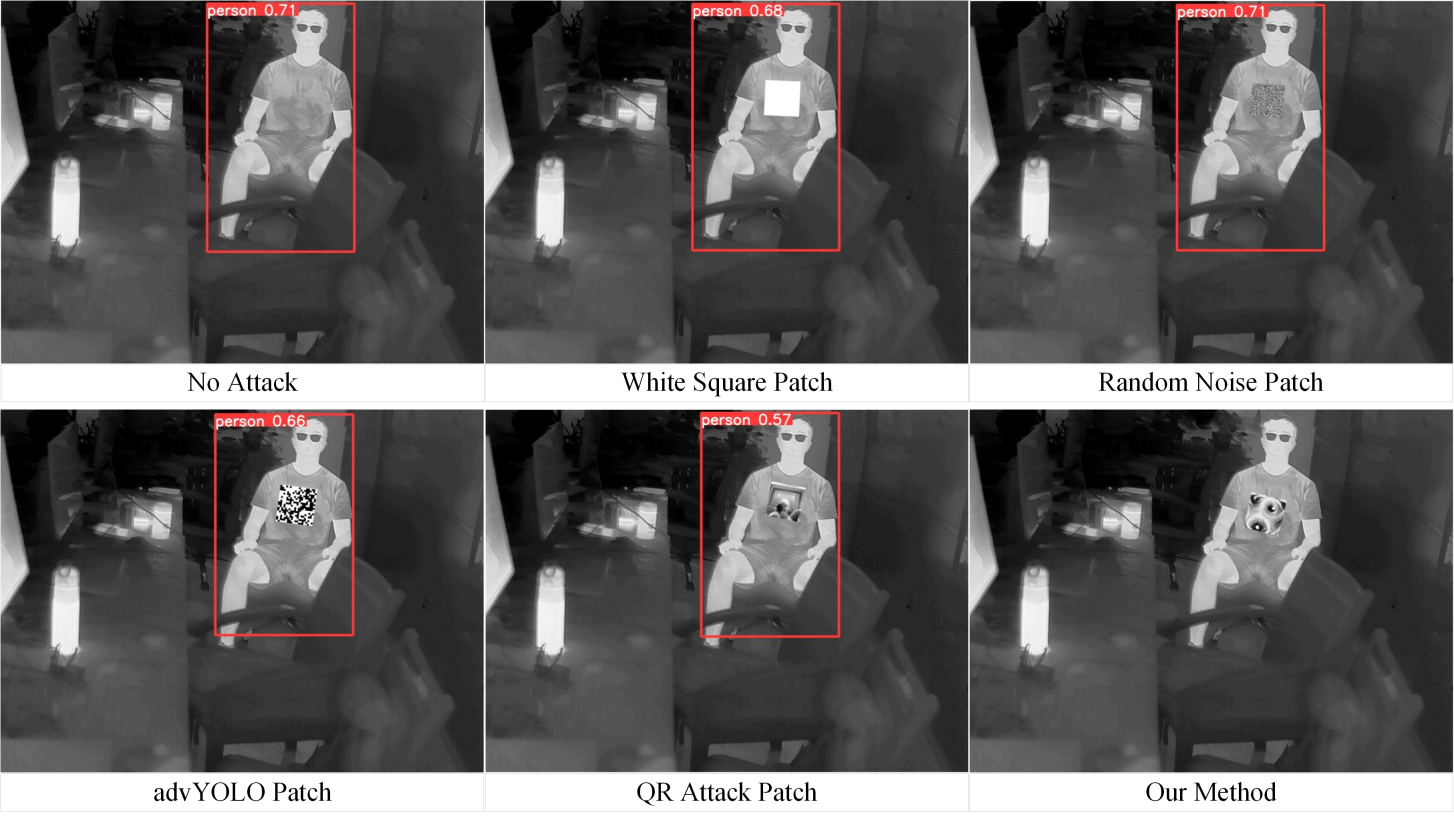
Comparison of the effectiveness of various adversarial attack methods.

To better assess the attack effectiveness of the GADP algorithm constructed in this study, five different scenarios were introduced for comparison: no attack, white square patch, random noise patch, advYOLO Patch, and QR Attack. Among these, the white square patch and random noise patch simulate scenarios where the target is partially occluded. The target recognition model used is YOLOv5s. Figure 16 presents one of the test results showing a successful attack.

In the absence of an attack, the confidence score of the “person” bounding box is 0.71. After applying the white square patch, QR Patch, and advYOLO Patch adversarial attack algorithms, the confidence score of the “person” decreases to 0.68, 0.66, and 0.57, respectively. The GADP algorithm proposed in this study achieves superior performance, causing YOLOv5s to fail in correctly detecting the “person.” The quantitative experimental results are summarized in Table 3.

The highest Attack Success Rate (ASR) is achieved by the proposed GADP algorithm, reaching 75.9%, followed by the QR Patch and advYOLO Patch, with success rates of 74.6% and 69.2%, respectively. In contrast, the success rates of the white square and random noise patches are below 9%. This is because data augmentation operations performed during the training of the infrared target recognition network already simulate the effects of occlusion and random noise. Consequently, such disturbances exert only a minor influence on the key structural information of infrared image targets. The loss function of the proposed GADP algorithm explicitly considers infrared image characteristics and optimizes the texture structure of the adversarial patch, resulting in a significant reduction in the Average Precision (AP) of the infrared target recognition model to 15.1%.

### 4.5. Adaptive Scaling of Patch Size and Performance Analysis

**Figure 17:**
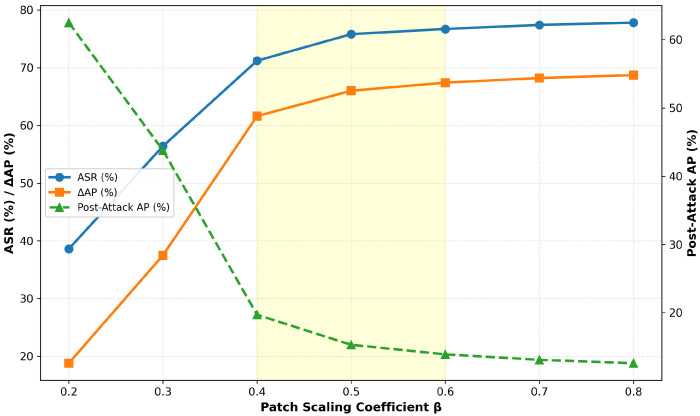
Impact of Patch Scaling Coefficient β on Attack Performance.

This section analyzes the influence of patch scaling during the application stage on the overall attack performance. As defined in Equation (43), the scaling coefficient β controls the relative coverage ratio of the adversarial patch with respect to the target. To verify the rationality of the range β∈[0.4,0.6], we compared the performance of different β values on the test set. Using the same pre-trained adversarial patch, we applied it to the test targets with β∈{0.3,0.4,0.5,0.6,0.7,0.8} and evaluated the detection performance of YOLOv5s. The results are illustrated in Figure 17.

From the trend of the curves, three key characteristics can be observed. First, when β<0.4, the Attack Success Rate (ASR) rises sharply with the increase in patch size, surging from 38.6% at β=0.2 to 71.2% at β=0.4. This indicates that, within this range, the patch coverage plays a decisive role in determining attack effectiveness. Second, within the interval β∈[0.4,0.6] (highlighted in yellow in the figure), the ASR curve becomes relatively stable, maintaining a high level between 71% and 77%, while the post-attack Average Precision (AP) drops below 20%. This demonstrates that this range achieves an optimal balance between attack strength and patch size. Finally, when β>0.6, a clear performance saturation effect appears: the ASR improvement narrows to within 1%, whereas the patch area continues to expand, resulting in diminishing marginal returns.

These results suggest that setting β=0.5 as the central value can maintain strong attack effectiveness (ASR = 75.8%) while keeping the patch area within 10% of the target size. This configuration satisfies the concealment requirement and avoids the computational redundancy caused by excessive optimization.

### 4.6. Transferability of Adversarial Attack Methods

In this experiment, four mainstream object detection models—YOLOv5s, YOLOv7, SSD, and Faster R-CNN—were selected to evaluate the transferability of the proposed adversarial attack algorithm. A total of four groups of experiments were conducted. This section first reveals the intrinsic mechanism of transferability through model similarity analysis, and then validates the transfer attack performance of the adversarial patches generated by the GADP algorithm through experimental results.

#### 4.6.1. Model Similarity Analysis

To quantitatively evaluate the similarity among different object detection models, the Kullback–Leibler (KL) divergence was adopted as a distance metric between models. The KL divergence measures the difference between two probability distributions and is defined as

(50) $$D_{KL}\left(P∥Q\right)=\sum_{i}P\left(i\right)\log\frac{P(i)}{Q(i)}$$

where *P* and *Q* denote the output probability distributions of two models on the same test dataset. Theoretically, a smaller KL divergence indicates higher similarity between the model outputs, implying better transferability of adversarial examples between the corresponding models.

We computed the pairwise KL divergence among the four object detection models on the test dataset, and the results are summarized in Table 4.

As shown in Table 4, the KL divergence between YOLOv5s and YOLOv7 is the smallest (0.18), which is consistent with expectations since both belong to the YOLO family of single-stage detectors and share similar detection paradigms and feature-extraction architectures. In contrast, the KL divergence between single-stage detectors (YOLOv5s, YOLOv7) and the two-stage detector (Faster R-CNN) is the largest (0.51–0.55), reflecting significant differences in output distributions arising from their distinct detection frameworks.

#### 4.6.2. Transfer Attack Experimental Results

In this experiment, four mainstream object detection models, YOLOv5s, YOLOv7, SSD, and Faster R-CNN, were selected to evaluate the transferability of adversarial attack algorithms, resulting in four groups of experiments. On the dataset used in this study, the Average Precisions for the category “person” achieved by YOLOv5s, YOLOv7, SSD, and Faster R-CNN were 81.3%, 88.3%, 70.8%, and 74.3%, respectively. At the same time, three adversarial attack algorithms, namely GADP, advYOLO Patch, and QR Attack, were compared.

In the first group of experiments, the adversarial patches were trained using YOLOv5s as the target detector, while the attacks were performed on multiple target detectors. The results are summarized in Table 5. As shown, the adversarial attack methods achieved the best performance against the YOLOv5s model. This is because the adversarial patches were trained jointly with the YOLOv5s model, leveraging knowledge of its network architecture, parameters, and weaknesses. The second most affected model was Faster R-CNN, whereas the SSD model showed the lowest vulnerability to transfer attacks. Moreover, the proposed GADP algorithm demonstrates slightly better performance in both attack effectiveness and transferability compared to advYOLO Patch and QR Attack. Specifically, GADP reduces the Average Precision of YOLOv5s to 14.8%, and in terms of transferability, it leads to a maximum reduction in Average Precision to 45.2% across the other three object detection models, achieving the best overall results.

The second set of experiments trained adversarial patches using the YOLOv7 model, and the results are presented in Table 6. The GADP algorithm achieved the best performance when attacking the YOLOv7 detector, reducing the AP for the “person” class to 11.5%. The YOLOv5s model ranked second, while the SSD model was the least affected. Since detectors in the YOLO series share similar network architectures and feature-extraction strategies, adversarial samples exhibit relatively strong transferability among them. The GADP algorithm reduced the AP of the other three object detection models by up to 35.8%, demonstrating superior attack effectiveness.

In the third set of experiments, adversarial patches were trained using the SSD model, as shown in Table 7. When attacking the SSD detector, the GADP algorithm achieved the most significant reduction in AP, decreasing it to 13.8%. The YOLOv5s detector followed, and the YOLOv7 detector was least affected. Across the other three object detection models, GADP reduced the AP by up to 44.9%, again showing the strongest attack performance.

The fourth set of experiments trained adversarial patches using the Faster R-CNN detector, with results summarized in Table 8. The GADP algorithm reduced the AP of the Faster R-CNN model to 10.1%, representing the best attack effectiveness. The SSD detector ranked second, and the YOLOv5s detector was least affected. For the other three object detection models, GADP reduced the AP by up to 38.1%, indicating strong adversarial transferability.

In summary, compared with advYOLO Patch and QR Attack, the GADP algorithm demonstrates superior transferability, reducing the Average Precision of common object detection models by approximately 40%. Moreover, adversarial patches generated by GADP also achieve strong white-box attack performance, lowering the AP of detectors with known gradients and network structures to around 11% and significantly degrading the detection performance of multiple different detectors.

Moreover, the transferability of adversarial samples shows no significant correlation with the KL divergence between models. For instance, in Table 5, the transfer performance of YOLOv5s against Faster R-CNN (KL divergence = 0.55, AP = 45.2%) is comparable to that against YOLOv7 (KL divergence = 0.18, AP = 45.3%). Similarly, in Table 7, the transfer attack from SSD to Faster R-CNN (KL divergence = 0.40, AP = 45.2%) even outperforms that to YOLOv7 (KL divergence = 0.30, AP = 47.3%).

This phenomenon may be attributed to the fact that KL divergence measures the difference in model output distributions on benign samples, whereas adversarial transferability is more likely governed by the gradient characteristics and the geometry of decision boundaries under specific perturbation directions. Furthermore, the GADP algorithm, through its multi-scale optimization strategy, may capture certain cross-architecture vulnerabilities inherent to object detection tasks. Therefore, a single distribution-based metric such as KL divergence is insufficient to accurately predict the transferability of adversarial samples.

## 5. Conclusions

In this study, we propose an adversarial patch-generation method for deep-learning-based infrared (IR) object detection tasks, aiming to enhance the robustness and transferability of adversarial patches. This approach demonstrates particular value in low-visibility scenarios, such as UAV detection and ground security. The proposed method effectively exposes the vulnerability of IR object detection systems by reducing the confidence of detected objects and misclassifying them as background.

The proposed GADP algorithm employs a dual-strategy optimization framework, incorporating Bernoulli random dropout and loss function constraints to improve the quality of generated adversarial patches. This design not only mitigates the risk of overfitting but also enhances the adaptability of patches across different object detection models, thereby improving their stealthiness and transferability. Experimental results demonstrate that the designed adversarial patches can successfully obscure multiple “person” instances in IR images, leading to a substantial reduction in detection rates of mainstream models while exhibiting strong transferability.

Furthermore, we integrate the Grad-CAM technique to locate key features in input images and adopt a random erasure strategy to improve the diversity of adversarial patches. The combination of these strategies enhances patch robustness under varying environmental conditions and viewing angles.

The research results provide a new perspective for understanding the security risks of deep learning models in infrared image processing, highlighting the necessity of continuously improving detection methods and defense strategies in infrared monitoring systems. Future research will explore additional enhancement strategies, real-world implementation challenges, and the evaluation of the proposed method across a broader range of deep learning models.

## Figures and Tables

**Table 1:** Comparison of adversarial attack algorithms.

Method	Advantages	Disadvantages
GAN-based adversarial attack methods	Good transferability; effective for image classification tasks	Training instability; slow convergence
Decision boundary-based adversarial attack methods	Fast computation; small perturbations	Difficult to formulate decision boundaries for complex models
Gradient optimization-based adversarial attacks	High success rate, simple implementation, suitable for white-box scenarios	Requires knowledge of the network’s gradient
Gradient-based adversarial attack methods	High Attack Success Rate; simple to implement	Requires access to model gradients; high computational cost
Patch-based adversarial attack methods	Easy to generate; efficient interference; practical for real-world deployment	Limited robustness

**Table 2:** System Performance of Partial Weight Parameters under Key Perturbation Factors.

Perturbation Setting	$\lambda_{1}$	$\lambda_{2}$	$\lambda_{3}$	$\lambda_{4}$	ASR (%)	SSIM	$L_{\mathrm{smooth}}$	$L_{\mathrm{pixel}}$
Baseline (1.0×)	22	0.4	5	2	73.2	0.66	15.2	2.58
$\lambda_{1}\times 0.5$	11	0.4	5	2	52.1	0.78	14.3	2.21
$\lambda_{1}\times 1.2$	26.4	0.4	5	2	76.8	0.61	15.9	2.85
$\lambda_{1}\times 1.5$	33	0.4	5	2	20.1	0.29	35.2	5.91
$\lambda_{2}\times 1.5$	22	0.6	5	2	24.3	0.84	4.5	2.42
$\lambda_{3}\times 0.5$	22	0.4	2.5	2	62.8	0.48	16.5	2.81
$\lambda_{3}\times 1.2$	22	0.4	6	2	74.9	0.74	14.7	2.49
$\lambda_{4}\times 0.5$	22	0.4	5	1	15.2	0.37	21.8	6.52
$\lambda_{4}\times 1.2$	22	0.4	5	2.4	74.5	0.68	14.8	2.04

**Table 3:** Comparison of effectiveness of adversarial Attack Methods.

Adversarial Attack Methods	Pre-Attack AP(%)	Post-Attack ASR (%)	Post-Attack AP (%)	ΔAP(%)
White Square Patch		6.5	75.6	5.7
Random Noise Patch		8.7	74.9	6.4
QR Patch	81.3	74.6	16.3	65.0
advYOLO Patch		69.2	16.8	64.5
GADP (Our Method)		75.9	15.1	66.2

**Table 4:** KL Divergence between Different Object Detection Models.

Model Combination	YOLOv5s vs. YOLOv7	YOLOv5s vs. SSD	YOLOv5s vs. Faster R-CNN	YOLOv7 vs. SSD	YOLOv7 vs. Faster R-CNN	SSD vs. Faster R-CNN
KL Divergence	0.18	0.32	0.55	0.30	0.51	0.40

**Table 5:** Adversarial Attack Performance of YOLOv5s-based Adversarial Samples.

Object Detection Models Used in Adversarial Patch Training	Adversarial Attack Methods	Post-Attack Average Precision (AP, %) of Object Detection Models			
		White-Box Adversarial Attack	Transferable Adversarial Attack		
		YOLOv5s	YOLOv7	SSD	Faster R-CNN
YOLOv5s	No Attack	81.3	88.3	70.8	74.3
	advYOLO Patch	15.6	47.4	49.6	46.1
	QR Patch	15.3	49.6	51.2	48.3
	GADP (Our Method)	14.8	45.3	49.1	45.2

**Table 6:** Adversarial Attack Performance of YOLOv7-based Adversarial Samples.

Object Detection Models Used in Adversarial Patch Training	Adversarial Attack Methods	Post-Attack Average Precision (AP, %) of Object Detection Models			
		White-Box Adversarial Attack	Transferable Adversarial Attack		
		YOLOv7	YOLOv5s	SSD	Faster R-CNN
YOLOv7	No Attack	88.3	81.3	70.8	74.3
	advYOLO Patch	13.7	37.6	39.1	40.5
	QR Patch	14.3	36.8	40.7	39.2
	GADP (Our Method)	11.5	35.8	38.2	37.4

**Table 7:** Adversarial Attack Performance of SSD-based Adversarial Samples.

Object Detection Models Used in Adversarial Patch Training	Adversarial Attack Methods	Post-Attack Average Precision (AP, %) of Object Detection Models			
		White-Box Adversarial Attack	Transferable Adversarial Attack		
		SSD	YOLOv5s	YOLOv7	Faster R-CNN
SSD	No Attack	70.8	81.3	88.3	74.3
	advYOLO Patch	14.7	46.3	50.4	46.6
	QR Patch	15.2	47.8	48.2	46.3
	GADP (Our Method)	13.8	44.9	47.3	45.2

**Table 8:** Adversarial Attack Performance of Faster R-CNN-based Adversarial Samples.

Object Detection Models Used in Adversarial Patch Training	Adversarial Attack Methods	Post-Attack Average Precision (AP, %) of Object Detection Models			
		White-Box Adversarial Attack	Transferable Adversarial Attack		
		Faster R-CNN	YOLOv5s	YOLOv7	SSD
Faster R-CNN	No Attack	74.3	81.3	88.3	70.8
	advYOLO Patch	11.4	45.3	41.8	40.5
	QR Patch	12.9	44.5	41.6	41.3
	GADP (Our Method)	10.1	43.7	40.	38.1

## Data Availability

The original contributions presented in this study are included in the article. Further inquiries can be directed to the corresponding authors.
